# Neuromuscular Function of the Knee Joint Following Knee Injuries: Does It Ever Get Back to Normal? A Systematic Review with Meta-Analyses

**DOI:** 10.1007/s40279-020-01386-6

**Published:** 2020-11-27

**Authors:** Beyza Tayfur, Chedsada Charuphongsa, Dylan Morrissey, Stuart Charles Miller

**Affiliations:** 1 Sports and Exercise Medicine, Queen Mary University of London, London, UK; 2 Physiotherapy Department, Barts Health NHS Trust, London, E1 4DG UK

## Abstract

**Background:**

Neuromuscular deficits are common following knee injuries and may contribute to early-onset post-traumatic osteoarthritis, likely mediated through quadriceps dysfunction.

**Objective:**

To identify how peri-articular neuromuscular function changes over time after knee injury and surgery.

**Design:**

Systematic review with meta-analyses.

**Data Sources:**

PubMed, Web of Science, Embase, Scopus, CENTRAL (Trials).

**Eligibility Criteria for Selecting Studies:**

Moderate and high-quality studies comparing neuromuscular function of muscles crossing the knee joint between a knee-injured population (ligamentous, meniscal, osteochondral lesions) and healthy controls. Outcomes included normalized isokinetic strength, muscle size, voluntary activation, cortical and spinal-reflex excitability, and other torque related outcomes.

**Results:**

A total of 46 studies of anterior cruciate ligament (ACL) and five of meniscal injury were included. For ACL injury, strength and voluntary activation deficits were evident (moderate to strong evidence). Cortical excitability was not affected at < 6 months (moderate evidence) but decreased at 24+ months (moderate evidence). Spinal-reflex excitability did not change at < 6 months (moderate evidence) but increased at 24+ months (strong evidence). We also found deficits in torque variability, rate of torque development, and electromechanical delay (very limited to moderate evidence). For meniscus injury, strength deficits were evident only in the short-term. No studies reported gastrocnemius, soleus or popliteus muscle outcomes for either injury. No studies were found for other ligamentous or chondral injuries.

**Conclusions:**

Neuromuscular deficits persist for years post-injury/surgery, though the majority of evidence is from ACL injured populations. Muscle strength deficits are accompanied by neural alterations and changes in control and timing of muscle force, but more studies are needed to fill the evidence gaps we have identified. Better characterisation and therapeutic strategies addressing these deficits could improve rehabilitation outcomes, and potentially prevent PTOA.

**Trial Registration Number:**

PROSPERO CRD42019141850.

**Electronic Supplementary Material:**

The online version of this article (10.1007/s40279-020-01386-6) contains supplementary material, which is available to authorized users.

## Key Points

**Table:** 

Neuromuscular alterations are evident in both short- and long-term following knee injuries in strength, voluntary activation, cortical and spinal excitability, and in timing and control of muscle force production.
These alterations may be specific to ACL injury, since we could not identify long-term alterations for meniscus injury and no studies could be found for other ligamentous or cartilage injuries to the knee, indicating a huge evidence gap.

## Introduction

Knee injury is an independent risk factor for the development of knee osteoarthritis (OA) in young adults [1–3]. The prevalence of post-traumatic OA (PTOA) can be as high as 80% at 10+ years after the initial injury [4], with 4–6 times higher odds compared to a non-injured knee [2]. PTOA mainly affects a younger and more active population when compared to non-traumatic OA, resulting in longer years lived with disability [5], and surgical interventions 7–9 years earlier in life [6]. Therefore, prevention strategies for PTOA development require particular attention.

Multiple anatomical, molecular, and physiological factors contribute to PTOA development [7]. Starting from the energy absorption at the time of trauma, damage to joint structures, including ligaments, meniscus, cartilage and subchondral bone singly or in combination, creates an inflammatory cycle. This cycle of activation of cartilage-degrading enzymes and chondrocyte apoptosis with joint instability and biomechanical alterations may further contribute to the degenerative process [7]. Throughout this process starting from the initial injury to PTOA initiation, it is important to identify modifiable risk factors so that targeted preventive rehabilitation strategies can be applied.

Muscles around the knee joint play an important role in the biomechanical alterations and joint instability after a knee injury. Quadriceps muscle weakness is a modifiable risk factor for non-traumatic OA [8] and PTOA [7]. Deficits in quadriceps strength are also common following knee injuries [9, 10], evident even at the end of the initial rehabilitation period [11], and may persist for more than 20 years [12]. Quadriceps weakness is also associated with gait alterations following knee injuries [13], which are common in the long-term [14], and hypothesised to be a contributor to PTOA initiation by abnormal knee cartilage loading [15]. These biomechanical alterations and joint instability may further contribute to the degenerative cycle within the knee joint [7]. Therefore, exercise therapy is at the core of PTOA prevention strategies to theoretically delay or prevent PTOA onset, through increasing muscle strength and improving neuromuscular function [16, 17].

While longitudinal data are available for quadriceps strength, less often considered is the overall neuromuscular function of the knee joint. Neuromuscular alterations after knee injury have been reported in case–control studies for strength [18], voluntary activation [19], cortical and spinal neural pathways [9, 10, 20], muscle structure [21] and muscle activation patterns [22, 23] in muscles including the quadriceps [9, 10, 18–21], hamstrings [22, 23] and gastrocnemii [23]. Knee joint loading is also not only determined by quadriceps femoris muscle but by the interaction of quadriceps, hamstrings, gastrocnemius and soleus muscles [24]. The neuromuscular alterations in these muscles controlling the knee joint may exacerbate the degenerative process after a knee injury through muscle weakness and abnormal cartilage loading [15]. It is therefore important to comprehensively understand neuromuscular alterations in all the muscles controlling the knee joint. This would further facilitate improved rehabilitation programs targeting these alterations.

Previous systematic reviews of this type of research typically considered isolated muscles, particular injuries, specific time-points or limited neuromuscular outcomes [19, 25–28]. There is a need to consider the importance of all injuries on all peri-articular knee muscles, the focus of this review, to fully understand the consequences of injury and possible links to PTOA. This review also aimed to identify where the main gaps in the literature manifest, so that future research and clinical recommendations can be optimally informed.

Injuries to knee ligaments, meniscus or cartilage are significantly associated with higher PTOA risk when compared to unspecified injuries [1–3, 29]. Therefore, the injured population should include ligament, meniscus and cartilage injuries to the knee if the aim is to understand the association with PTOA development. There is also evidence of bilateral neuromuscular changes following unilateral knee injury [30, 31], suggesting a requirement for healthy control groups instead of using the contralateral ‘healthy leg’ for an unbiased evaluation of post-traumatic neuromuscular alterations. Therefore, we aimed to determine how neuromuscular function of the knee joint changes over time following knee injuries involving ligament, meniscus or cartilage compared to healthy controls.

## Methods

This systematic review and meta-analysis complied with the PRISMA (Preferred Reporting Items for Systematic Reviews and Meta-Analyses) guidelines [32]. The study protocol was registered on PROSPERO (International Prospective Register of Systematic Reviews) (CRD42019141850, 25 July 2019).

### Search Strategy

We conducted a comprehensive systematic search of the following electronic databases without date restrictions until February 2020: PubMed, Embase, Web of Science, Scopus, and Cochrane Central Register of Controlled Trials (CENTRAL). The search terms included medical subject headings (MeSH) terms and text words. We modified the search strategy for each specific database with keywords and concepts remaining identical. The main concept included (knee injury [anterior cruciate ligament (ACL), posterior cruciate ligament (PCL), medial collateral ligament (MCL), lateral collateral ligament (LCL), meniscus, cartilage, chondral] AND neuromuscular [strength, reflex, activation, electromyography, size] AND lower limb muscles [quadriceps, hamstring, gastrocnemius, soleus, popliteus]). Search strategies for all databases can be found in Electronic Supplementary Material Appendix S1. Two reviewers (BT and CC) independently conducted the searches, removed duplicates, screened all abstracts for eligibility and retrieved full-text versions of the eligible articles. Disagreements between reviewer’s judgements were resolved with a third reviewer (SCM). We also searched the reference lists of the included articles and of the systematic reviews for additional studies.

### Selection Criteria

Studies comparing neuromuscular function of the knee joint in participants with a previous knee injury and/or knee surgery (all ligamentous, meniscal, osteochondral lesions) to an age- and sex-matched control group were eligible for inclusion. Studies without a control group, comparing involved limb to uninvolved limb of participants, were excluded, as there is evidence of bilateral neuromuscular changes following unilateral injury [30, 31]. Observational studies both with cross-sectional or prospective designs and interventional studies were included. We only used the baseline data of interventional studies. Only studies published in the English language were included.

### Outcome Measures

Studies had to report at least one of the following neuromuscular outcome measures as the main outcome to be included: body-mass normalized muscle strength as measured by an isokinetic dynamometer or fixed force transducer, torque related outcomes such as rate of torque development, torque variability or electromechanical delay, muscle size or volume, voluntary activation deficits as measured by central activation ratio or twitch interpolation technique, spinal reflex excitability, or corticomotor excitability as measured by active motor threshold. We defined neuromuscular as including muscle size or volume, spinal reflex excitability and corticomotor excitability although we are aware that others may define it as outcomes specifically related to the force-generating capacity of the muscles.

### Methodological Quality Assessment

Risk of bias of the included studies was assessed using a modified version of the Downs and Black checklist [33, 34], a methodological quality assessment tool for both randomised and non-randomised interventional studies with high internal consistency and inter-rater reliability [33]. The modified version consists of 15 questions, excluding the questions about randomisation and interventions from the original version (Electronic Supplementary Material Appendix S2). The highest score of the modified version is 16, and thresholds for low, moderate and high quality were accepted as < 60% (≤ 9), 60–74% (10–11), and > 75% (≥ 12), respectively, consistent with previous studies [14, 35]. We excluded low-quality studies from this systematic review as they may cause over- or under-estimation of effect sizes and may distort results, therefore leading to incorrect conclusions [36, 37]. Two independent reviewers (BT and CC) assessed methodological quality and disagreements were resolved by asking a third reviewer (SCM).

### Data Extraction

Data regarding the study design, participant characteristics (number of participants, age, sex, injury/surgery details, time since injury/surgery) and outcome measures (measured muscle groups and outcome) were extracted by two independent reviewers (BT and CC) in an Excel spreadsheet. Disagreements were resolved by asking a third reviewer (SCM). Group means and standard deviations were extracted for the main outcome measures. Where the reported data were insufficient, corresponding authors were contacted by e-mail to request unreported data or additional details.

### Data Analysis

We analysed data according to time since injury/surgery, consistent with previous systematic reviews [14, 27], as follows: (1) less than 6 months (< 6 months); (2) 6 months to less than 12 months (6–12 months); (3) 12 months to less than 2 years (1–2 years); and (4) 2 years and over (≥ 2 years). When pre-surgery data were reported in surgical treatment papers, time since injury was used to determine the time subgroup of pre-surgery data and time since surgery was used for the post-surgery data.

Data were pooled for meta-analysis when there were more than two studies reporting the same outcome measure, using the Cochrane Review Manager software (Version 5.3. Copenhagen: The Nordic Cochrane Centre, the Cochrane Collaboration, 2014). Standardised mean differences (SMD; Hedges’ adjusted *g*) with 95% confidence intervals (CIs) were calculated for variables of interest as the difference between the injured leg and healthy control leg. Heterogeneity of the pooled data was analysed with *I*^2^ and was considered as no heterogeneity (≤ 25%), low heterogeneity (> 25%), moderate heterogeneity (> 50%), and high heterogeneity (> 75%) [38]. We used fixed (for homogenous data, *I*^2^ ≤ 25%) or random (for heterogeneous data, *I*^2^ > 25%) effects models for each meta-analysis according to the statistical heterogeneity. The magnitude of the pooled SMD was interpreted based on Cohen’s criteria, where SMD ≥ 0.8 indicated large, 0.5–0.8 moderate, and 0.2–0.5 small effect sizes [39]. Potential publication biases were also examined by funnel plots for meta-analyses when 10 or more studies were included [37]. Level of evidence was reported by the following criteria: strong evidence (multiple high-quality studies that were statistically homogenous); moderate evidence (multiple studies including at least one high-quality study, or from multiple moderate-quality studies that are statistically homogenous); limited evidence (high-quality study or multiple moderate-quality studies that are statistically heterogeneous); very limited evidence (one moderate-quality) [40].

We also provided an evidence gap map, showing the level of evidence of available literature with findings, and areas that need further research. This aims to avoid research waste in areas with strong evidence and guide future studies.

## Results

### Study Selection

**Fig. 1:**
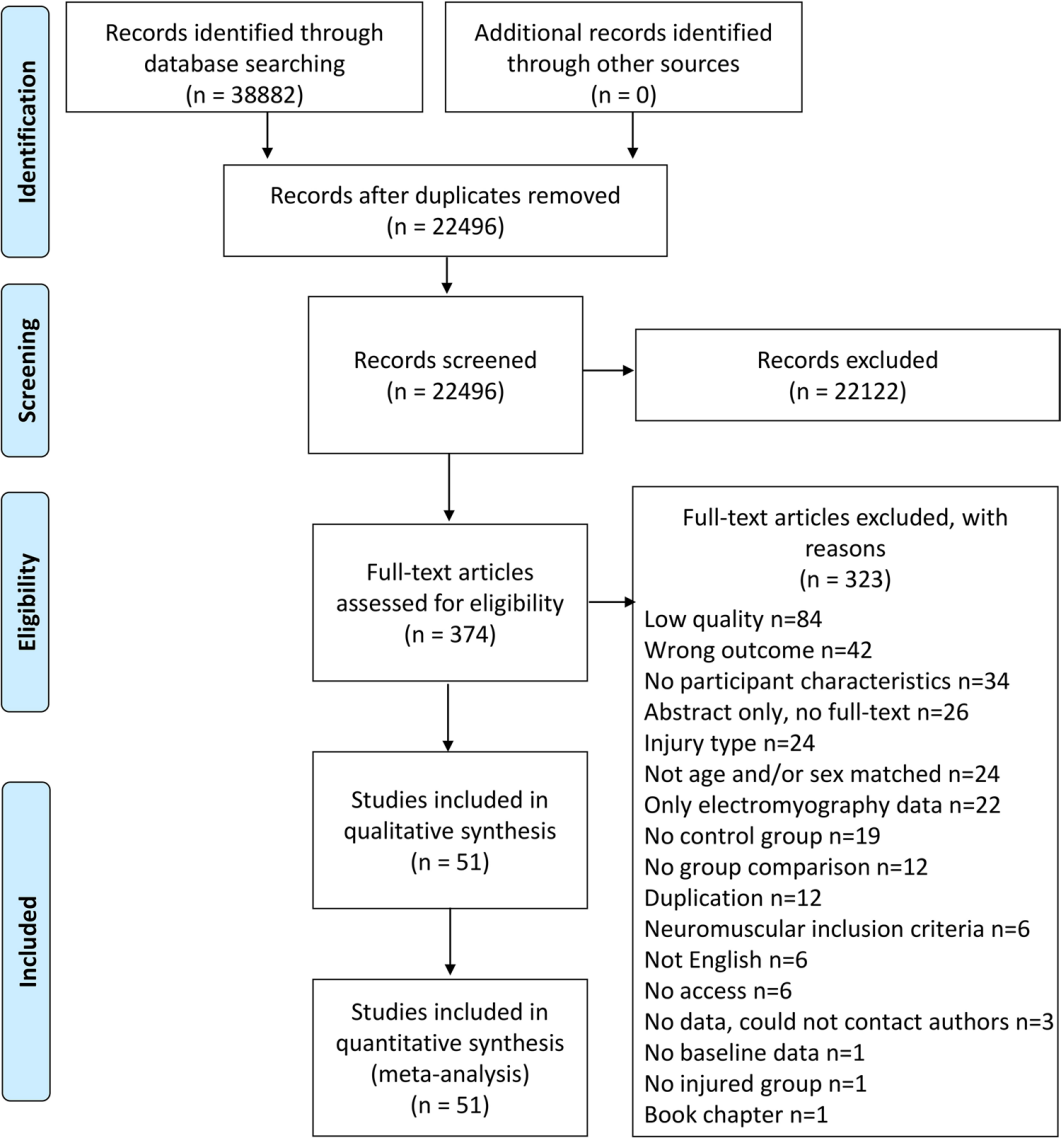
Flow diagram of the study selection process

The search strategy retrieved 22,496 papers after duplicate removal (Fig. 1). Following title and abstract screening, 374 articles were assessed in full-text and 137 studies were eligible to undergo quality assessment.

Following quality assessment, 84 low-quality studies were excluded, leaving 13 high-quality (HQ) and 38 moderate-quality (MQ) studies for final inclusion. Details of methodological quality assessment of included studies can be found in Table 1 and of excluded studies in Electronic Supplementary Material Appendix S3.

**Table 1:** Methodological quality assessment of included studies based on a modified Downs and Black scale [33, 34]

Study	1	2	3	5	6	7	10	11	12	15	18	20	21	22	25	Total score	Quality level
Almeida et al. [41]	1	1	1	1	1	1	1	1	0	0	1	1	1	0	0	11	M
Chung et al. [42]	1	1	1	2	1	1	1	1	0	0	1	1	1	0	1	13	H
Clagg et al. [43]	1	1	1	1	1	1	1	0	0	0	1	1	1	0	0	10	M
Engelen-van Melick et al. [44]	1	1	1	2	1	1	0	1	0	0	1	1	0	0	1	11	M
Freddolini et al. [45]	1	1	1	2	1	1	1	0	0	0	1	1	0	0	1	11	M
Garrison et al. [46]	1	1	1	1	1	1	1	0	0	0	1	1	0	0	1	10	M
Goetschius and Hart [47]	1	1	1	2	1	1	1	0	0	0	1	1	0	0	0	10	M
Goetschius et al. [48]	1	1	1	2	1	1	1	0	0	0	1	1	1	0	1	12	H
Hall et al. [49]	1	1	1	1	1	1	1	0	0	0	1	1	1	0	0	10	M
Harkey et al. [10]	1	1	1	2	1	1	1	0	0	0	1	1	1	0	1	12	H
Holsgaard-Larsen et al. [50]	1	1	1	2	1	1	1	0	0	0	1	1	1	1	1	13	H
Hsiao et al. [51]	1	1	1	1	1	1	0	1	1	0	1	1	1	0	0	11	M
Hsieh et al. [52]	1	1	1	1	1	1	1	0	0	0	1	1	1	0	0	10	M
Ilich et al. [53]	1	1	1	2	1	1	1	0	0	0	1	1	1	0	0	11	M
Johnson et al. [54]	1	1	1	2	1	1	1	0	0	0	1	1	0	0	0	10	M
Kaminska et al. [55]	1	1	1	1	1	1	1	0	0	0	1	1	1	0	0	10	M
Kellis et al. [56]	1	1	1	1	1	1	0	1	0	0	1	1	1	0	0	10	M
Kline et al. [57]	1	1	1	1	1	1	1	0	0	0	1	1	1	0	0	10	M
Krishnan and Williams [58]	1	1	1	2	1	1	1	0	0	0	1	1	0	0	1	11	M
Kuenze et al. [20]	1	1	1	2	1	1	0	1	0	0	1	1	1	0	1	12	H
Kuenze et al. [59]	1	1	1	2	1	1	1	0	0	0	1	1	1	1	1	13	H
Kvist et al. [60]	1	1	1	1	1	1	1	1	0	0	1	1	0	0	0	10	M
Larsen et al. [11]	1	1	1	2	1	1	0	1	0	0	1	1	1	0	0	11	M
Lepley et al. [61]	1	1	1	2	1	1	1	0	0	0	1	1	1	0	1	12	H
Lepley et al. [9]	1	1	1	2	1	1	1	0	0	0	1	1	1	0	1	12	H
Lepley et al. [62]	1	1	1	2	1	1	1	0	0	0	1	1	0	0	1	11	M
Maeda et al. [63]	1	1	1	2	1	1	1	0	0	0	1	1	0	0	1	11	M
Norte et al. [64]	1	1	1	2	1	1	1	0	0	0	1	1	1	0	0	11	M
Oeffinger et al. [65]	1	1	1	1	1	1	1	1	0	0	1	1	0	0	0	10	M
O’Malley et al. [66]	1	1	1	1	1	1	1	1	0	0	1	1	0	0	0	10	M
Pamukoff et al. [22]	1	1	1	2	1	1	1	0	0	0	1	1	0	0	1	11	M
Pamukoff et al. [67]	1	1	1	2	1	1	1	0	0	0	1	1	0	0	1	11	M
Reed-Jones and Vallis [68]	1	1	1	1	1	1	1	0	0	0	1	1	1	0	0	10	M
Ristanis et al. [69]	1	1	1	2	1	1	1	0	0	0	1	1	0	0	1	11	M
Roos et al. [70]	1	1	1	2	1	1	1	0	0	0	1	1	0	0	0	10	M
Scheurer et al. [71]	1	1	1	2	1	1	1	0	0	0	1	1	0	0	1	11	M
Sturnieks et al. [72]	1	1	1	2	1	1	1	0	0	0	1	1	0	0	1	11	M
Tengman et al. [12]	1	1	1	2	1	1	1	1	0	0	1	1	1	0	1	13	H
Thomas et al. [73]	1	1	1	2	1	1	1	0	0	0	1	1	0	0	1	11	M
Thorlund et al. [74]	1	1	1	2	1	1	1	1	0	0	1	1	1	1	0	13	H
Thorlund et al. [75]	1	1	1	1	1	1	1	1	1	0	1	1	1	0	0	12	H
Tourville et al. [76]	1	1	1	1	1	1	1	0	0	0	1	1	1	1	1	12	H
Tsarouhas et al. [77]	1	1	1	1	1	1	1	1	0	0	1	1	0	0	0	10	M
Vairo [78]	1	1	1	2	1	1	1	1	0	0	1	1	0	0	1	12	H
Vairo et al. [79]	1	1	1	2	1	1	1	0	0	0	1	1	0	0	1	11	M
Ward et al. [80]	1	1	1	1	1	1	1	1	0	0	1	1	1	0	0	11	M
Welling et al. [81]	1	1	1	1	1	1	1	0	0	0	1	1	1	0	0	10	M
Xergia et al. [82]	1	1	1	2	1	1	1	0	0	0	1	1	0	0	0	10	M
Zarzycki et al. [83]	1	1	1	1	1	1	1	0	0	0	1	1	0	0	1	10	M
Zult et al. [84]	1	1	1	2	1	1	1	0	0	0	1	1	0	0	1	11	M
Zwolski et al. [85]	1	1	1	1	1	1	1	0	0	0	1	1	1	0	1	11	M

Numbers in the top row are the item numbers in the original Downs and Black scale

*H* high quality, *M* moderate quality

### Study Characteristics

The characteristics of the included studies and outcome measures in each study can be found in Electronic Supplementary Material Appendix S4. Overall, 46 studies included patients with ACL injury and five studies included patients with a meniscus injury. ACL studies included patients with ACL deficient knees and ACL reconstruction patients with different graft types (i.e. hamstring tendon graft (HT), patellar tendon graft (PT), allograft), while all meniscus studies included patients who had had a meniscectomy. ACL studies included a younger population (i.e. participants in their 20 s) when compared to meniscus studies (i.e. participants in their 40 s) at the time of testing. We could not identify any studies including patients with other ligamentous injuries to the knee (i.e. PCL, MCL, and LCL) or cartilage/chondral injuries as isolated injuries. In addition, studies generally tested quadriceps and hamstring muscles, with no studies reporting any outcomes pertaining to the gastrocnemius, soleus or popliteus muscles.

### Findings

Initial meta-analyses showed that injury type caused large heterogeneity in the pooled data (i.e. opposing direction of effects based on injury type). Therefore, we performed our meta-analyses for studies of ACL and meniscus injury separately. ACL-deficient and ACL-reconstructed cohorts yielded similar results and did not cause heterogeneity; therefore, they were pooled together in all meta-analyses.

**Fig. 2:**
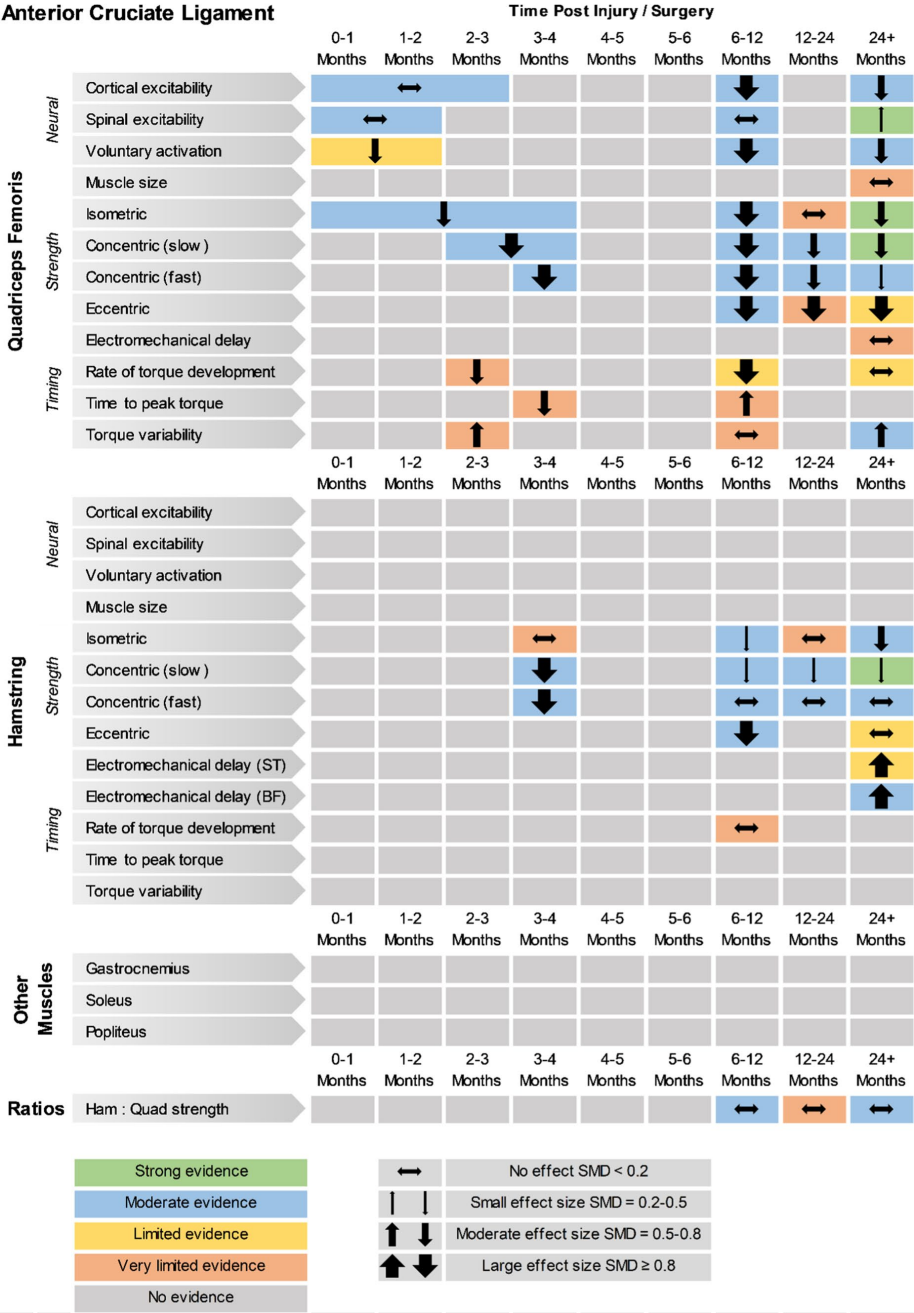
Findings and literature gap map for anterior cruciate ligament studies. Colours represent the evidence level as by van Tulder et al. [40] and directions represent injured group data when compared to control, with the effect size. *SMD* standardised mean difference, *ST* semitendinosus, *BF* biceps femoris, *Ham:Quad* hamstring:quadriceps

**Fig. 3:**
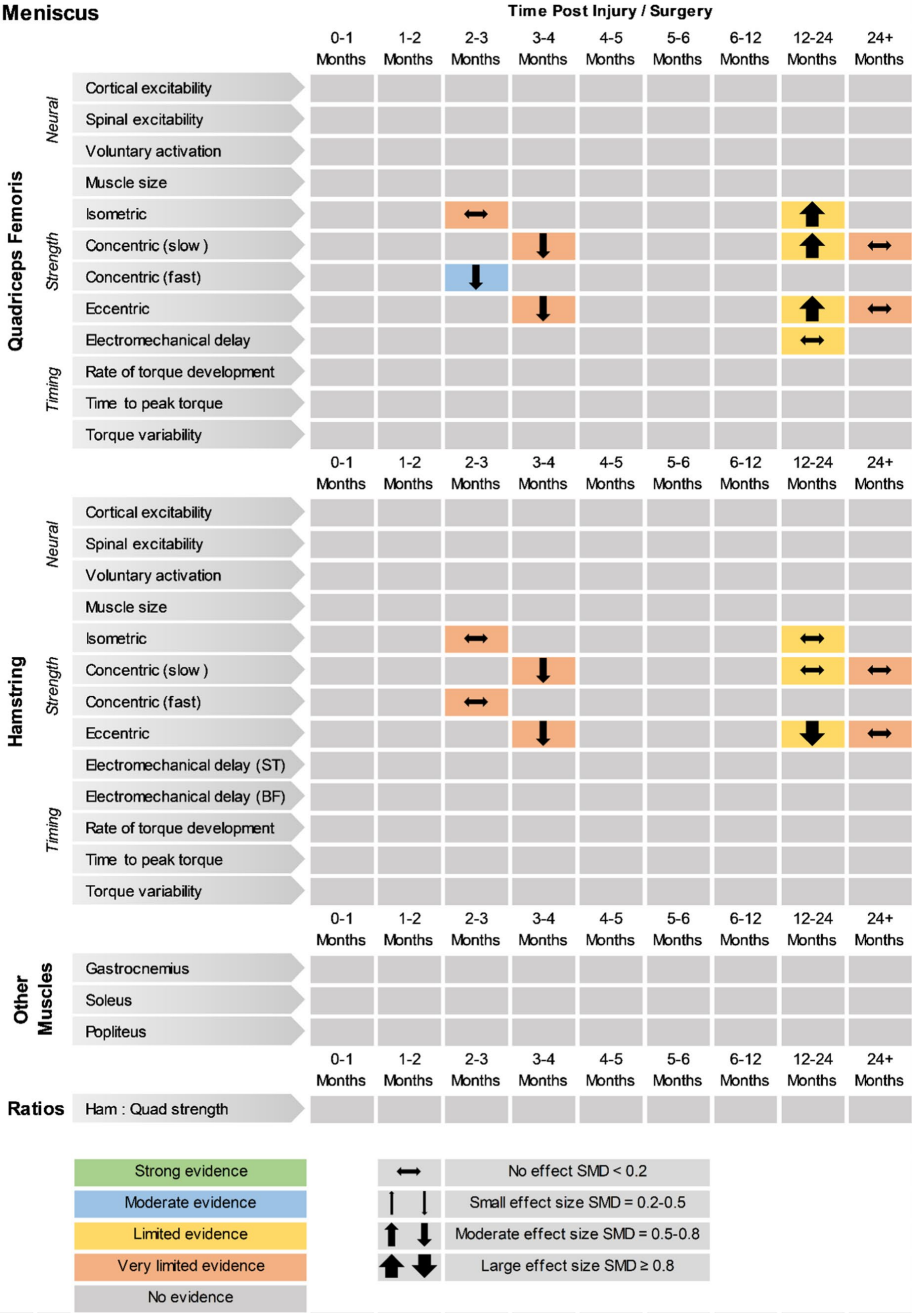
Findings and literature gap map for meniscus studies. Colours represent the evidence level as by van Tulder et al. [40] and directions represent injured group data when compared to control, with the effect size. *SMD* standardised mean difference, *ST* semitendinosus, *BF* biceps femoris, *Ham:Quad* hamstring:quadriceps

**Fig. 4:**
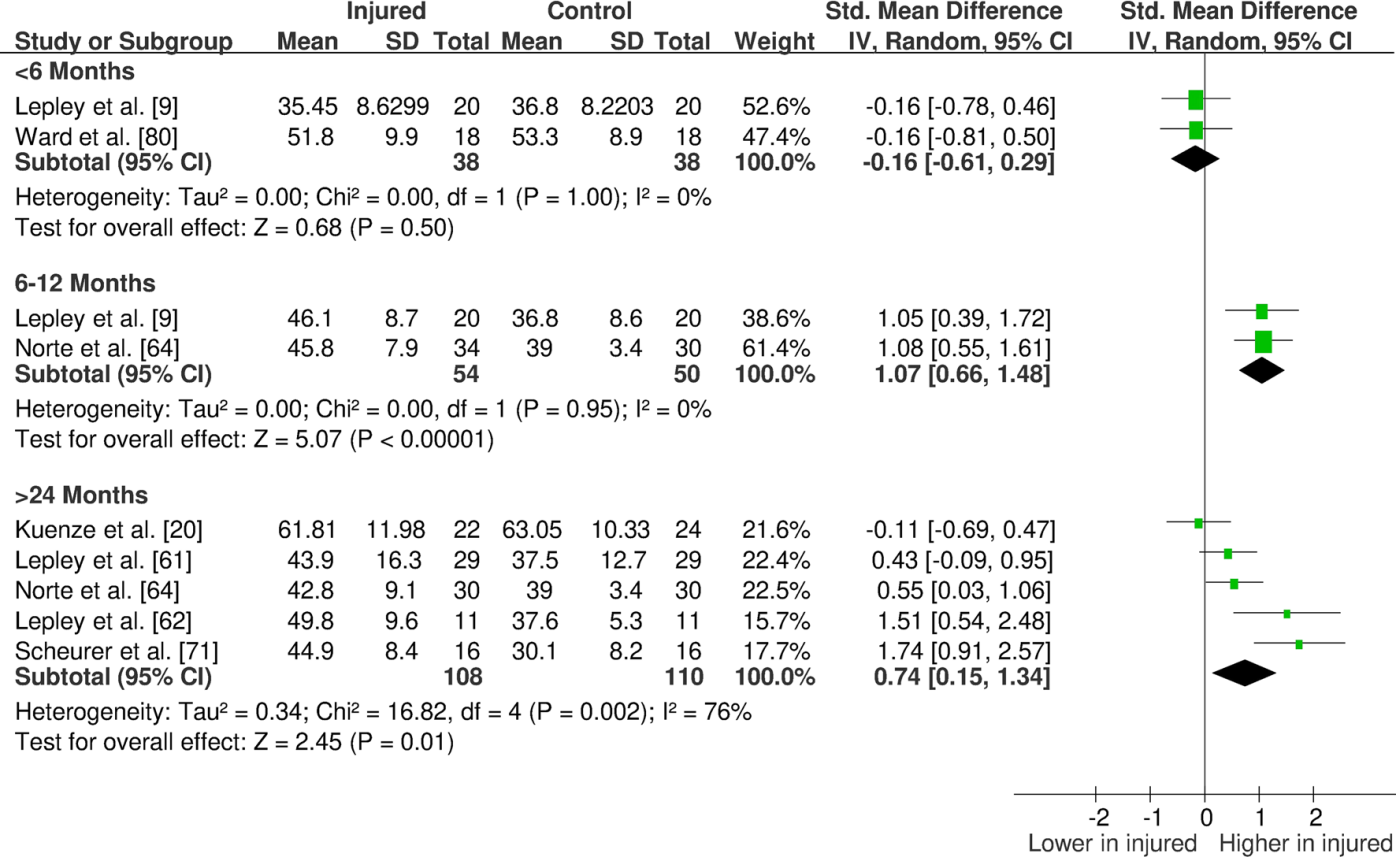
Forest plot of quadriceps active motor threshold from anterior cruciate ligament studies (increased active motor threshold meaning decreased cortical excitability)

**Fig. 5:**
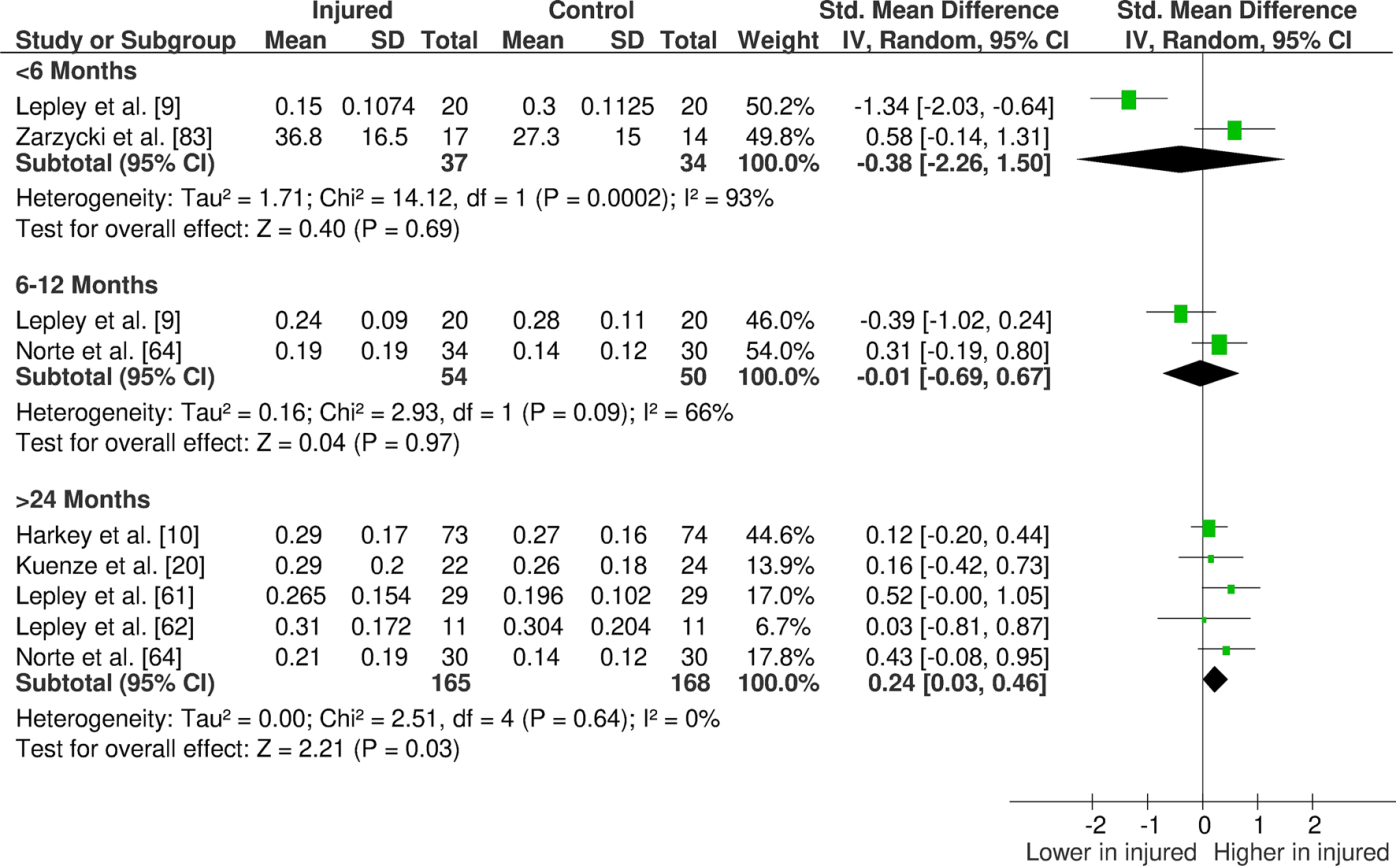
Forest plot of quadriceps Hoffman reflex (spinal excitability) from anterior cruciate ligament studies

**Fig. 6:**
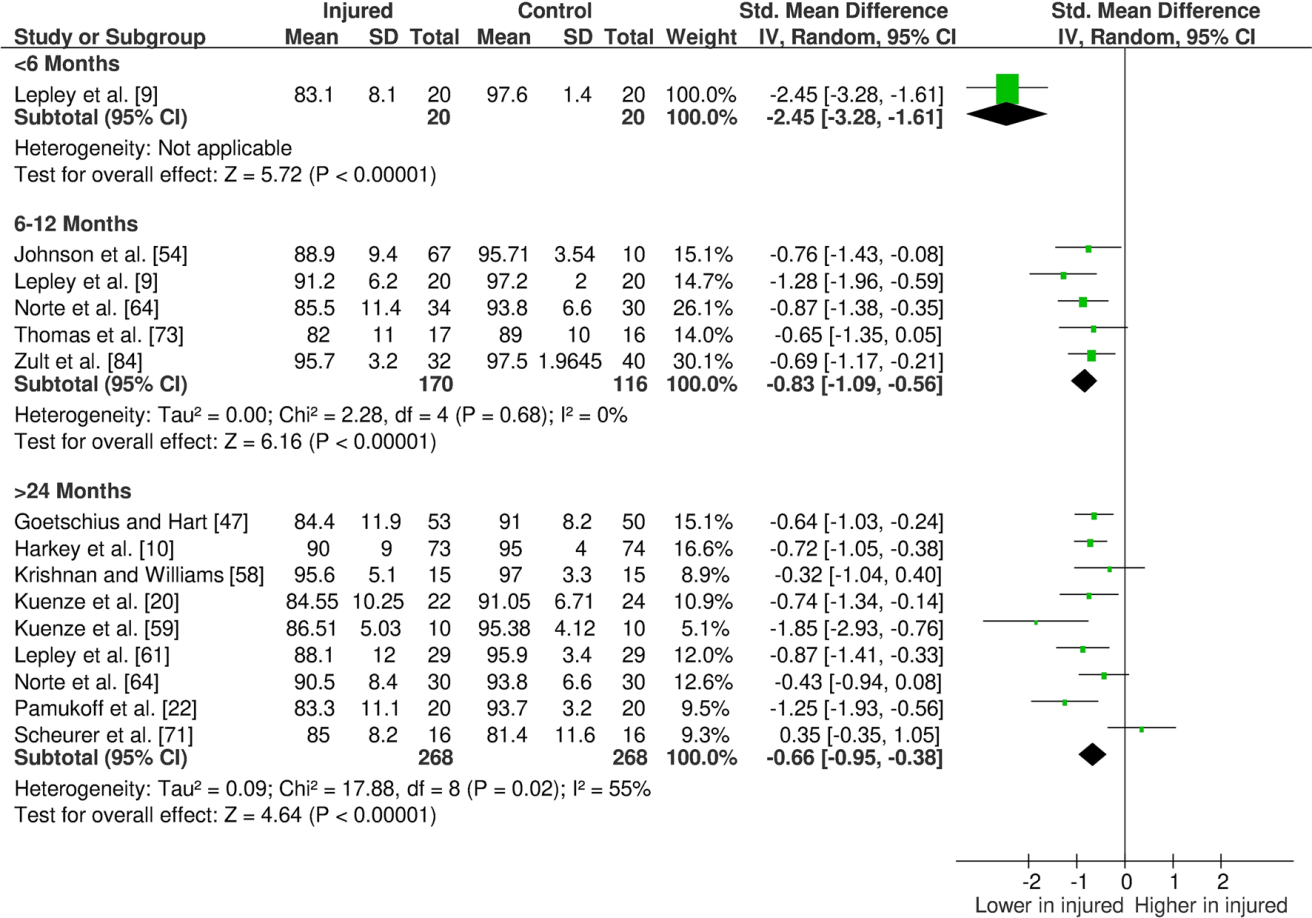
Forest plot of quadriceps voluntary activation from anterior cruciate ligament studies

**Fig. 7:**
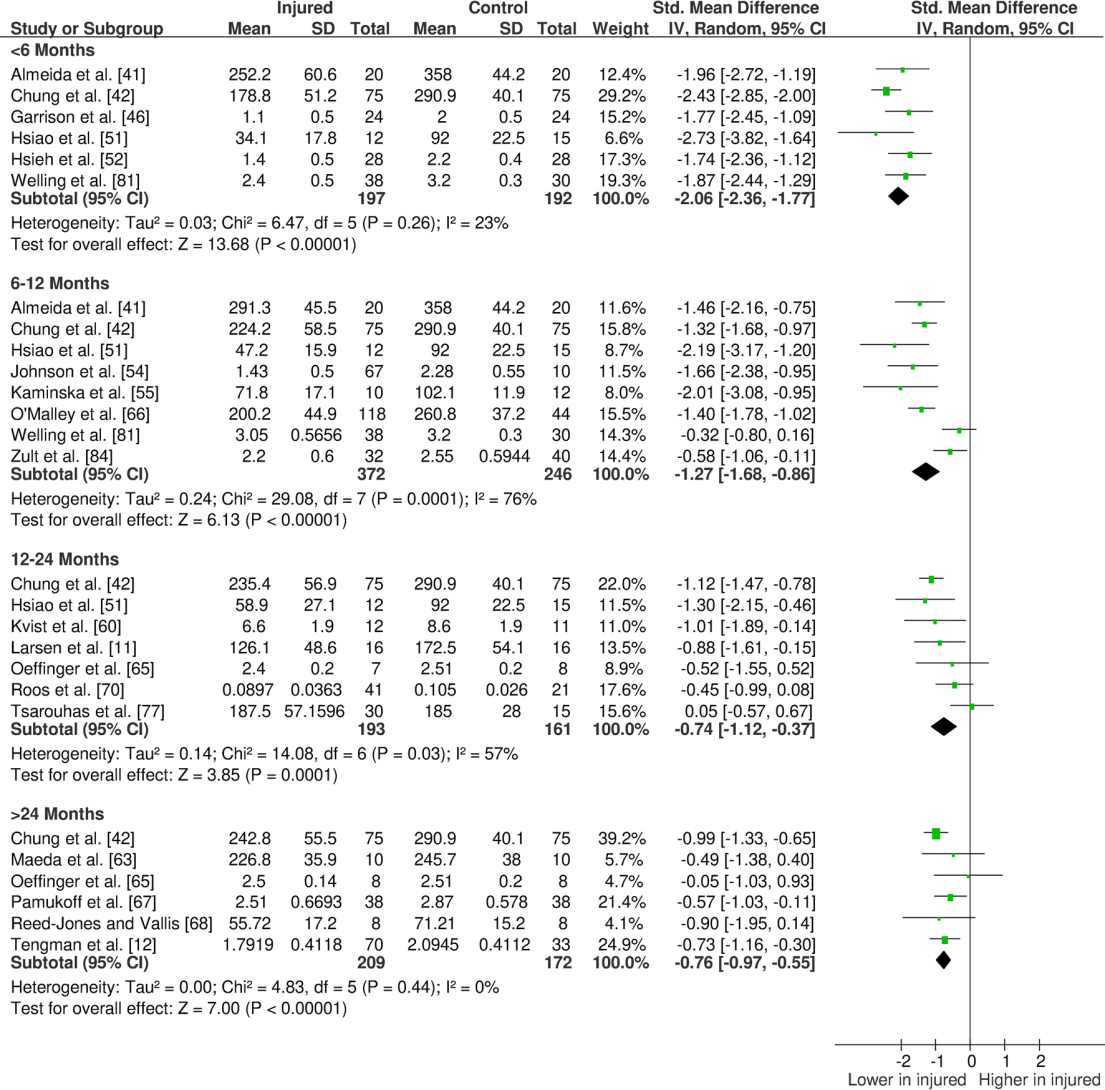
Forest plot of quadriceps slow concentric strength from anterior cruciate ligament studies

**Fig. 8:**
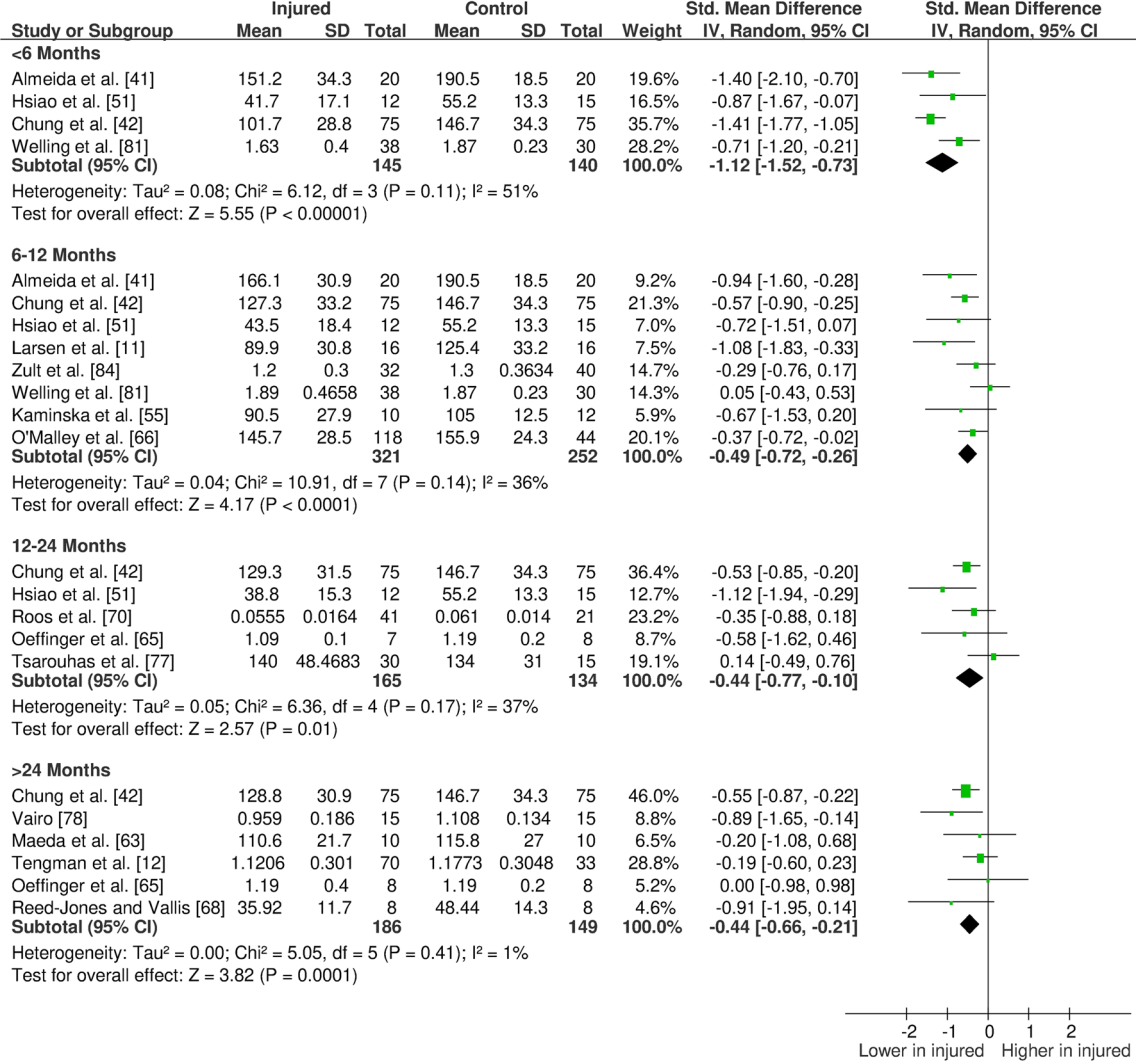
Forest plot of hamstring slow concentric strength from anterior cruciate ligament studies

The overall findings (direction, effect size and level of evidence) of all meta-analyses for each outcome measure for the given time period post-injury/surgery were summarised in evidence gap maps (Fig. 2 for ACL studies and Fig. 3 for meniscus studies). We broke down the first 6 months in more detail to show the evidence gap for the post-injury rehabilitation period. However, the data for the first 6 months are pooled together in the meta-analyses and the gap map is only showing which months the data are derived from. We could not identify any publication bias for eligible outcomes (i.e. with more than ten studies in the meta-analysis; quadriceps isometric strength) as measured by funnel plots. The forest plots for quadriceps cortical excitability (Fig. 4), quadriceps spinal excitability (Fig. 5), quadriceps voluntary activation (Fig. 6), quadriceps slow concentric strength (Fig. 7), and hamstring slow concentric strength (Fig. 8) for ACL studies are presented. All other meta-analyses, forest plots and funnel plots can be found in Electronic Supplementary Material Appendix S5.

Our results showed consistent quadriceps and hamstring strength deficits in both the short- and long-term after ACL injury/surgery regardless of contraction type (i.e. isometric, concentric or eccentric) with moderate and strong evidence. These deficits were in parallel to voluntary activation deficits in the short- (limited evidence) and long-term (moderate evidence). Cortical and spinal excitability were not affected in the short-term (moderate evidence); however, they were altered in the long-term differently. Cortical excitability decreased in the long-term (moderate evidence), while spinal excitability increased (strong evidence). Muscle size was reported in only one study, providing very limited evidence of no long-term change. Other findings for the quadriceps femoris muscle for patients with ACL injury/surgery included decreased rate of torque development (limited to very limited evidence), decreased (< 6 months) then increased (6–12 months) time to peak torque (very limited evidence), increased torque variability (very limited to moderate evidence), and unaffected electromechanical delay (very limited evidence). Additionally, hamstring rate of torque development was not affected (very limited evidence); however, electromechanical delay increased in the long-term (limited to moderate evidence). No change was seen in hamstring to quadriceps strength ratios (very limited to moderate evidence).

Meniscus studies reported quadriceps and hamstring strength deficits in the short-term (i.e. the first 6 months after injury/surgery), with quadriceps strength greater than controls in the second year following injury/surgery, and similar to controls in the long-term (i.e. 24+ months post injury/surgery), albeit with limited or very limited evidence. Also, no change was reported for quadriceps rate of torque development in the long-term (limited evidence). Other neuromuscular outcomes for meniscus injuries have not been investigated, leaving a huge evidence gap for this voluminous patient population.

## Discussion

Neuromuscular alterations around the knee joint are commonly reported following knee injuries but remain poorly understood due to lack of adequate synthesis. The aim of this systematic review was to identify changes in neuromuscular function of the knee joint over time following knee injury/surgery. Central and peripheral neural changes, morphological muscle changes, and the clinical manifestations of altered amplitude and timing of muscle activation and torque control were included in the analysis to provide a comprehensive overview. The timeline of these changes was also provided, enabling the comparison of short- and long-term changes after injury. Following ACL injuries, we found evidence for deficits in quadriceps and hamstring strength and quadriceps voluntary activation, changes in cortical and spinal-reflex neural pathways, deficits in force control and delays in rapid force generation post-injury. Following meniscus injuries, there was limited evidence for immediate strength deficits, with these being restored long-term. Importantly, we identified major gaps in the evidence base, with no studies on patients with cartilage injuries or ligamentous injuries other than ACL, and no studies measuring gastrocnemius, soleus or popliteus muscles for any of the injuries.

We consistently found quadriceps and hamstring strength deficits in the ACL injured group both in the short- and long-term. Increasing muscle strength is a primary focus of rehabilitation guidelines [86–88]; however, impairments are evident despite these efforts. We also found that quadriceps voluntary activation deficits are evident in the short-term and do not recover in the long-term, providing a potential underlying neural mechanism of the quadriceps muscle weakness. This neural dysfunction, often described as arthrogenic muscle inhibition (AMI), is hypothesised to be a protective mechanism to avoid further joint damage following knee injuries [89]. However, it can be problematic if not restored through rehabilitation, which would appear to be the case for most of the participants measured in the included studies. We could not control for the effects of rehabilitation received post-injury and, therefore, cannot comment on whether AMI persistence is mediated by the appropriateness of a particular rehabilitation approach. A recent scoping review suggested the use of cryotherapy and exercise in the management of AMI, albeit partly based on experimentally induced AMI in healthy knees [90]. It was also shown that after ACLR, a 2-week rehabilitation programme including cryotherapy application and physical exercise together improves AMI more than cryotherapy or exercise alone [91]. Currently, exercise treatment is accepted as common practice [86–88], and our meta-analysis of 14 studies (Fig. 6) showed a lack of activation deficit resolution in the long-term, suggesting either the rehabilitation approaches undertaken by the recruited participants in the included studies were insufficient for resolving these deficits, adherence was sub-optimal or the implementation of rehabilitation strategies were lacking.

Quadriceps muscle strength and voluntary activation deficits were evident at the time return to sport commonly occurs (i.e. 6–12 months post-injury/surgery). Current rehabilitation and return to sport guidelines recommend a limb symmetry index threshold of 85–90% as a criterion for strength recovery [86–88, 92, 93]. However, the presence of neuromuscular alterations in the contralateral limb may cause overestimation of the injured-limb function [30, 31]. As such, use of symmetry-based strength outcomes may reduce the ability to detect the strength deficits we found in this systematic review, as we did not accept contralateral knee as a control group. It may be that identification of normative ranges from future research would better inform return-to-play decisions.

We aimed to understand the nature of relevant central and peripheral nervous system changes, including cortical and spinal-reflexive pathways and found that these change with time. We found no change in cortical excitability or in spinal-reflex excitability in the short-term, with moderate evidence. Short-term swelling and pain may be present following knee injury/surgery, which does not affect cortical excitability but decreases spinal-reflex excitability [94, 95]. Experimental joint effusion studies have also shown that effusion decreases spinal-reflex excitability immediately after injection [96, 97]. Therefore, the observed acute unaffected values of spinal-reflex excitability may be due to both swelling and pain shadowing an increased spinal reflex excitability in the short-term.

In the longer term, there is strong evidence of decreased cortical excitability and increased spinal-reflex excitability, suggesting that neuromodulation of quadriceps activation adapts and changes through time after injury/surgery. Decreased cortical excitability means that knee-injured patients need more stimulation to yield sufficient excitation in the primary motor cortex to generate muscle activation [98]. While the clinical importance of these changes in corticospinal and spinal-reflexive pathways is not fully understood, recently it has been shown that corticospinal adaptations are correlated with muscle strength and patient-reported knee function satisfaction following ACLR [99]. It may be that the decrease in cortical excitability is a protective long-term motor cortex adaptation, while a compensatory reflex mechanism maintains required muscle function when needed i.e. as a preparatory mechanism to avoid a sudden collapse of the knee joint in knee-injured patients [100]. It has been suggested that electromyographic biofeedback, transcranial magnetic stimulation or transcutaneous electrical nerve stimulation may be beneficial in changing neural pathways to improve muscle function [101]; however, empirical data are lacking to support these recommendations [90]. Further studies exploring the effects of different interventions on neuromodulation of quadriceps may be helpful to understand the clinical usefulness of these, or novel, modalities and approaches.

Meniscus injury caused heterogeneity and showed better long-term outcomes when compared to ACL studies. Altered sensory function is reported following ACL injuries and has been hypothesised to be the cause of alterations in motor response [102]. Our results provide enough evidence to support that these changes in neuromuscular function are seen in ACL-injured patients. However, it should be noted that included studies were not investigating only isolated ACL injury effects as many of the ACL-injured participants had a concomitant meniscal injury. Due to a lack of reporting in most studies, we could not pool or detail the differences between isolated ACL injuries vs those with concomitant meniscus damage. There is evidence that combined injuries may increase PTOA development risk when compared to isolated ACL injuries [103, 104]; however, from the neuromuscular perspective, no difference was reported in quadriceps strength or voluntary activation for isolated ACL injuries vs ACL injuries with concomitant meniscus injury [105]. Therefore, neuromuscular alterations, mediated through quadriceps weakness, may not be a critical pathway towards PTOA onset in patients with isolated meniscus injuries, although the number of studies included in this study was insufficient to draw a conclusion. We speculate that our findings show preliminary data supporting injury-specific changes, and should stimulate further investigation in injury-specific groups, perhaps grouping injuries into cogent sub-groups.

There is a decreased rate of quadriceps torque development, albeit with limited evidence, which would limit rapid force production in knee-injured populations [106, 107]. This may be due to an increased neural processing time or a delay in the transmission of force within the muscle and/or tendon [108, 109]. Rapid force production may be more relevant to daily life activities and sports than maximum strength, as most of these activities require a quick muscle response [106, 107]. Rapid force production is also correlated with self-reported knee function [52] and functional performance [110], and may not recover even if maximum peak torque is regained [107]. Therefore, the rate of torque development may be an important descriptor of muscle function and further attention should be given to strengthen the evidence and clarify the clinical relevance.

There is moderate evidence that quadriceps torque variability increases in the long-term, suggesting muscle control impairments. Precise control of movement is essential for optimal knee function, and insufficiency may cause alterations in joint loading which may, in turn, lead to degenerative cartilage changes [111]. Increased torque variability is also evident in knee OA patients [112]; thus, we speculate that motor control of the quadriceps muscle may be another component of neuromuscular alterations in the long-term following injury/surgery potentially contributing to the initiation of knee OA.

We found an important evidence gap in the literature concerning the change of muscle morphology. Our search yielded several studies on muscle size; however, they either did not include a suitable control group or were of low quality; due to procedural and reporting issues rather than the absence of valid measurement tools. Only one moderate quality study, providing very limited evidence, found no difference in quadriceps muscle volume in the long-term. However, it has been reported that both neural alterations and muscle size can predict up to 60% of the variance in muscle strength post-injury [113]. Muscle atrophy may also explain strength deficits more than activation failure [114]. Muscle size may have played an important role in the strength deficits found in this systematic review; therefore, future studies should consider measuring muscle size in knee-injured populations together with other neuromuscular outcomes.

Future research is needed to improve our understanding of neuromuscular changes post-injury, with morphological and neural alterations being measured in the same knee-injured populations to understand their interactions and effects on muscle strength, as well as muscle control and the timing of movement generation. Another future step should be understanding the impact of these neuromuscular alterations on movement patterns and joint loading, and therefore their potential implications for PTOA onset. We suggest including structural measurement of OA presence in knee-injured populations to understand possible associations of neuromuscular alterations with OA presence. A clear association would further inform prospective studies to determine whether these associations are causal.

Despite the increasing number of publications, and accepted functional importance [115], we still do not have strong evidence for key short- and long-term neuromuscular outcomes post-injury. The main research focus has been on muscle strength, while the underlying neural mechanism or morphological changes within the muscle have been of less interest. More data are required to determine changes in neuromuscular outcomes such as muscle size, timing of muscle force production (i.e. rate of torque development, electromechanical delay) and force control (i.e. torque variability). While the importance of measuring these factors in the quadriceps muscle, especially post-ACL injury, is well-established, changes in other muscles are often neglected and should be further investigated.

Understanding the effects of different interventions may help the development of better rehabilitation protocols that may address the persistent neuromuscular impairments we have shown in our systematic review. Future studies should consider repeated measurement of neuromuscular function to better understand its relation to changes in the patient’s reported outcome measures and function. Further, such data may yield useful findings about the prognostic value of neuromuscular functional measures, which could help guide both optimised rehabilitation and detection of osteoarthritis development, while explaining individual differences in responses. Therefore, the effects of novel rehabilitation strategies that target neuromuscular alterations of the knee joint in knee-injured populations should be investigated and further implemented in rehabilitation protocols to improve short- and long-term outcomes.

### Limitations and Considerations for Future Studies

Studies systematically lacked reporting of participant selection procedures, possibly resulting in a high level of participant selection bias (Table 1). Included patients may be those still having symptoms in the long-term, which may result in an inflated alteration in the injured group. Ideally, recruitment would be as close as possible to the index injury, with long-term follow-up, so as to include those who cope well with injury as well as those who do not (i.e. a prospective study design).

There is evidence of altered neuro-muscular function in the lower limb being a risk factor for knee injury [116]. Therefore, we cannot assume that identified deficits are purely the result of the injury as they may have been predisposing factors to injury in the first place.

Our search strategy included terms of all knee ligaments (ACL, PCL, MCL, and LCL), meniscal injuries, cartilage injuries and post-traumatic OA. However, our results only showed studies on ACL-injured or meniscus-injured populations. We also found that knee injury was a source of heterogeneity in some of the outcomes; therefore, our findings may be specific to ACL and meniscus injuries, and not applicable to other injuries.

Another limitation was the heterogeneity of the included patients in most studies. ACL-injured patients included in the studies were mixed both in terms of concomitant injury, ‘copers’ vs ‘non-copers’, and graft type if surgery was performed. Due to a lack of reporting and inadequate study numbers, we could not draw any conclusions on the effects of concomitant injuries or different surgeries (i.e. different grafts), or comparison of ‘copers’ with ‘non-copers’.

Time since injury/surgery was used to define the short- and long-term changes and for grouping the studies to pool the data. Many of the included studies were not strict in their time since injury/surgery criteria; therefore, the variability was high. I.e. a study could include participants with a time since injury/surgery from 6 to 60 months, with a median of 24 months. We used the mean or median time to define the time groups; therefore, some variability in the data may be expected due to the heterogeneity of time since injury/surgery ranges of the included participants.

### Clinical Implications

Persistent deficits found in our study may highlight possible failures in current post-injury treatment strategies. We found that quadriceps strength, voluntary activation, control and speed of muscle force generation and hamstring strength are affected; therefore, targeting these deficits may improve functional outcomes of knee-injury rehabilitation. We acknowledge that measuring most of the neuromuscular outcomes reported in this study may not be feasible in clinical practice (i.e. cortical excitability, spinal reflexes, torque-related outcomes, etc.); however, research shows clinically applicable rehabilitation strategies may improve these outcomes. For example, strength training alone may not be sufficient to improve neuromuscular function of the knee joint, if movement quality and speed of force production are being overlooked. It has been suggested that a training protocol including controlled muscle contractions with low-loads may improve muscle force control [117], and heavy- or explosive-type resistance training [106], or sensorimotor training focusing on postural stabilization [118] may improve the rate of torque development. For strength recovery, cryotherapy combined with physical exercise has been shown to be effective in reducing muscle inhibition in the short-term after injury [90], while progressive strength training shows promising results in the long-term [81]. Implementing these different exercise types may improve neuromuscular function of the knee joint, thus enhancing functional outcome post-injury with repeated measures of neuromuscular function potentially useful to determine intervention mechanisms alongside clinical effectiveness. Such information could inform more detailed rules for return to physical activity/sport criteria, such as including motor control and quality of movement as well as maximum force capacity of muscles. The subsequent effect on PTOA development or re-injury rates would be key impact markers. It should be noted that our findings are mainly based on ACL-injured populations; therefore future studies may yield different results for different injury types (i.e. injuries to the other ligaments in the knee joint, meniscus or cartilage).

## Conclusion

Our study enhances understanding of neuromuscular function of the knee joint following injuries and shows that neural and muscular alterations are common and persistent in the short- and long-term after injury/surgery. Strength and voluntary activation deficits are accompanied by changes in cortical and spinal excitability for ACL patients in both the short- and long-term (moderate to strong evidence), as well as deficits in force control and rapid force production (very limited to moderate evidence). Only strength was investigated in patients with meniscus injuries and short-term deficits demonstrated. Our study facilitates clinical recognition of these deficits, and promotes future research to advance rehabilitation strategies to target these alterations, ultimately contributing to efforts made to optimise clinical outcomes following knee injury and/or surgery and minimise PTOA development or re-injury.

## Electronic Supplementary Material

Below is the link to the electronic supplementary material.

Supplementary file1 (DOC 18427 KB)

## Declarations

### Availability of data and material

The data that support the findings of this study are available on request from the corresponding author [BT].
