# Supplementary material for: Neuromuscular Function of the Knee Joint Following Knee Injuries: Does It Ever Get Back to Normal? A Systematic Review with Meta-Analyses
Source: Sports Med. 2020 Nov 27;51(2):321–38. doi: 10.1007/s40279-020-01386-6 (PMC7846527; doi:10.1007/s40279-020-01386-6)
Supplement: Supplementary file 1 — Supplementary file1 (DOC 18427 KB) [file 40279_2020_1386_MOESM1_ESM.doc]

**Title: Neuromuscular function of the knee joint following knee injuries: Does it ever get back to normal? A systematic review with meta-analyses**

**Journal: Sports Medicine**

Authors: Beyza Tayfur^1^, Chedsada Charuphongsa^1^, Dylan Morrissey^1,2^, Stuart Charles Miller^1^

^1^Sports and Exercise Medicine, Queen Mary University of London, London, United Kingdom

^2^Physiotherapy department, Barts Health NHS trust, London E1 4DG

Corresponding Author:

Beyza Tayfur

Sports and Exercise Medicine, First Floor, Mile End Hospital, Bancroft Road, London E1 4DG

+447460733817

[b.tayfur@qmul.ac.uk](mailto:b.tayfur@qmul.ac.uk)

**Electronic Supplementary Material Appendix S1. Search Strategy for All Databases**

**Search performed on 04.02.2020.**

**Pubmed :** ("Knee Injuries"[Mesh] OR "Posterior Cruciate Ligament"[Mesh] OR "Posterior Cruciate Ligament Reconstruction"[Mesh] OR "Meniscus"[Mesh] OR "Tibial Meniscus Injuries"[Mesh] OR "Menisci, Tibial"[Mesh] OR "Anterior Cruciate Ligament"[Mesh] OR "Anterior Cruciate Ligament Injuries"[Mesh] OR "Medial Collateral Ligament, Knee"[Mesh] OR Knee injur* OR knee surg* OR ACL OR anterior cruciate ligament OR menisc* OR flap tear* OR bucket handle tear* OR medial collateral ligament OR MCL OR Lateral collateral ligament OR LCL OR cartilage* OR chondral OR PCL OR posterior cruciate ligament* OR cruciate ligament)

AND

("Muscle Strength"[Mesh] OR "Electromyography"[Mesh] OR "Reflex, Monosynaptic"[Mesh] OR "H-Reflex"[Mesh] OR "Muscle Contraction"[Mesh] OR neurophysiolog* OR neuromuscular OR physiolog* OR morpholog* OR mass OR volume OR composition OR cross-sectional area OR voluntar* OR strength OR weakness OR atrophy OR power OR force OR performance OR innervation OR denervation OR activation OR activity OR inhibition OR function OR dysfunction OR electromyograph* OR EMG OR myograph* OR reflex* OR excitability OR excitation OR contraction* OR H-reflex* OR M-wave OR electromechanic*)

AND

("Quadriceps Muscle"[Mesh] OR "Hamstring Muscles"[Mesh] OR knee extens* OR quadriceps OR lower limb MUSCLE* OR rectus femoris OR vastus lateralis OR vastus intermedius OR vastus medialis OR knee flex* OR hamstring* OR biceps femoris OR semitendinosus OR semimembranosus OR GASTROCNEMIUS OR SOLEUS OR CALF MUSCLE* OR POPLITEUS OR ankle plantar flex*)

**Web of Science Search:**

(("Knee Injuries"[Mesh] OR "Posterior Cruciate Ligament"[Mesh] OR "Posterior Cruciate Ligament Reconstruction"[Mesh] OR "Meniscus"[Mesh] OR "Tibial Meniscus Injuries"[Mesh] OR "Menisci, Tibial"[Mesh] OR "Anterior Cruciate Ligament"[Mesh] OR "Anterior Cruciate Ligament Injuries"[Mesh] OR "Medial Collateral Ligament, Knee"[Mesh] OR Knee injur* OR knee surg* OR ACL OR anterior cruciate ligament OR menisc* OR flap tear* OR bucket handle tear* OR medial collateral ligament OR MCL OR Lateral collateral ligament OR LCL OR cartilage* OR chondral OR PCL OR posterior cruciate ligament* OR cruciate ligament) AND ("Muscle Strength"[Mesh] OR "Electromyography"[Mesh] OR "Reflex, Monosynaptic"[Mesh] OR "H-Reflex"[Mesh] OR "Muscle Contraction"[Mesh] OR neurophysiolog* OR neuromuscular OR physiolog* OR morpholog* OR mass OR volume OR composition OR cross-sectional area OR voluntar* OR strength OR weakness OR atrophy OR power OR force OR performance OR innervation OR denervation OR activation OR activity OR inhibition OR function OR dysfunction OR electromyograph* OR EMG OR myograph* OR reflex* OR excitability OR excitation OR contraction* OR H-reflex* OR M-wave OR electromechanic*) AND ("Quadriceps Muscle"[Mesh] OR "Hamstring Muscles"[Mesh] OR knee extens* OR quadriceps OR lower limb muscle* OR rectus femoris OR vastus lateralis OR vastus intermedius OR vastus medialis OR knee flex* OR hamstring* OR biceps femoris OR semitendinosus OR semimembranosus OR gastrocnemius OR soleus OR calf muscle* OR popliteus OR ankle plantar flex*))

Additional Filters; Human Studies (for Pubmed)

No language restrictions.

Sorted by ‘Most Recent’ in Pubmed.

Title abstract search in Pubmed.

Topic search in Web of Science.

**Embase:**

(**'knee injur*'**:ti,ab,kw OR **'knee surg*'**:ti,ab,kw OR **'acl'**:ti,ab,kw OR **'anterior cruciate ligament'**:ti,ab,kw OR **'menisc*'**:ti,ab,kw OR **'flap tear*'**:ti,ab,kw OR **'bucket handle tear*'**:ti,ab,kw OR **'medial collateral ligament'**:ti,ab,kw OR **'mcl'**:ti,ab,kw OR **'lateral collateral ligament'**:ti,ab,kw OR **'lcl'**:ti,ab,kw OR **'cartilage*'**:ti,ab,kw OR **'chondral'**:ti,ab,kw OR **'pcl'**:ti,ab,kw OR **'posterior cruciate ligament*'**:ti,ab,kw OR **'cruciate ligament'**:ti,ab,kw) AND (**'neurophysiolog*'**:ti,ab,kw OR **'neuromuscular'**:ti,ab,kw OR **'physiolog*'**:ti,ab,kw OR **'morpholog*'**:ti,ab,kw OR **'mass'**:ti,ab,kw OR **'volume'**:ti,ab,kw OR **'composition'**:ti,ab,kw OR **'cross-sectional area'**:ti,ab,kw OR **'voluntar*'**:ti,ab,kw OR **'strength'**:ti,ab,kw OR **'weakness'**:ti,ab,kw OR **'atrophy'**:ti,ab,kw OR **'power'**:ti,ab,kw OR **'force'**:ti,ab,kw OR **'performance'**:ti,ab,kw OR **'innervation'**:ti,ab,kw OR **'denervation'**:ti,ab,kw OR **'activation'**:ti,ab,kw OR **'activity'**:ti,ab,kw OR **'inhibition'**:ti,ab,kw OR **'function'**:ti,ab,kw OR **'dysfunction'**:ti,ab,kw OR **'electromyograph*'**:ti,ab,kw OR **'emg'**:ti,ab,kw OR **'myograph*'**:ti,ab,kw OR **'reflex*'**:ti,ab,kw OR **'excitability'**:ti,ab,kw OR **'excitation'**:ti,ab,kw OR **'contraction*'**:ti,ab,kw OR **'h-reflex*'**:ti,ab,kw OR **'m-wave'**:ti,ab,kw OR **'electromechanic*'**:ti,ab,kw) AND (**'knee extens*'**:ti,ab,kw OR **'quadriceps'**:ti,ab,kw OR **'lower limb muscle*'**:ti,ab,kw OR **'rectus femoris'**:ti,ab,kw OR **'vastus lateralis'**:ti,ab,kw OR **'vastus intermedius'**:ti,ab,kw OR **'vastus medialis'**:ti,ab,kw OR **'knee flex*'**:ti,ab,kw OR **'hamstring*'**:ti,ab,kw OR **'biceps femoris'**:ti,ab,kw OR **'semitendinosus'**:ti,ab,kw OR **'semimembranosus'**:ti,ab,kw OR **'gastrocnemius'**:ti,ab,kw OR **'soleus'**:ti,ab,kw OR **'calf muscle*'**:ti,ab,kw OR **'popliteus'**:ti,ab,kw OR **'ankle plantar flex*'**:ti,ab,kw) AND **'human'**/de

Title, abstract, keywords search.

## Scopus Search:

Title, abstract, keywords search.

TITLE-ABS-KEY ( ( knee-injur*  OR  knee-surg*  OR  acl  OR  anterior-cruciate-ligament  OR  menisc*  OR  flap-tear*  OR  bucket-handle-tear*  OR  medial-collateral-ligament  OR  mcl  OR  lateral-collateral-ligament  OR  lcl  OR  cartilage*  OR  chondral  OR  pcl  OR  posterior-cruciate-ligament*  OR  cruciate-ligament )

AND

 ( neurophysiolog*  OR  neuromuscular  OR  physiolog*  OR  morpholog*  OR  mass  OR  volume  OR  composition  OR  cross-sectional-area  OR  voluntar*  OR  strength  OR  weakness  OR  atrophy  OR  power  OR  force  OR  performance  OR  innervation  OR  denervation  OR  activation  OR  activity  OR  inhibition  OR  function  OR  dysfunction  OR  electromyograph*  OR  emg  OR  myograph*  OR  reflex*  OR  excitability  OR  excitation  OR  contraction*  OR  h-reflex*  OR  m-wave  OR  electromechanic* )

AND

 ( knee-extens*  OR  quadriceps  OR  lower-limb-muscle*  OR  rectus-femoris  OR  vastus-lateralis  OR  vastus-intermedius  OR  vastus-medialis  OR  knee-flex*  OR  hamstring*  OR  biceps-femoris  OR  semitendinosus  OR  semimembranosus  OR  gastrocnemius  OR  soleus  OR  calf-muscle*  OR  popliteus OR ankle-plantar-flex* )

AND

 ( LIMIT-TO ( EXACTKEYWORD ,  "Human" ) )

**Central (Trials) Search:**

'**Knee injur* OR knee surg* OR ACL OR anterior cruciate ligament OR menisc* OR flap tear* OR bucket handle tear* OR medial collateral ligament OR MCL OR Lateral collateral ligament OR LCL OR cartilage* OR chondral OR PCL OR posterior cruciate ligament* OR cruciate ligament in Title, Abstract, Keywords**

**and**

**neurophysiolog* OR neuromuscular OR physiolog* OR morpholog* OR mass OR volume OR composition OR cross-sectional area OR voluntar* OR strength OR weakness OR atrophy OR power OR force OR performance OR innervation OR denervation OR activation OR activity OR inhibition OR function OR dysfunction OR electromyograph* OR EMG OR myograph* OR reflex* OR excitability OR excitation OR contraction* OR H-reflex* OR M-wave OR electromechanic* in Title, Abstract, Keywords**

**and**

**knee extens* OR quadriceps OR lower limb MUSCLE* OR rectus femoris OR vastus lateralis OR vastus intermedius OR vastus medialis OR knee flex* OR hamstring* OR biceps femoris OR semitendinosus OR semimembranosus OR GASTROCNEMIUS OR SOLEUS OR CALF MUSCLE* OR POPLITEUS** OR **ankle plantar flex* in Title, Abstract, Keywords**in **Trials**'

RESULTS:

PUBMED: 7431

WOS: 14748

EMBASE: 5745

CENTRAL: 3433

SCOPUS: 7525

OVERALL: 38882

**Electronic Supplementary Material Appendix S2. Risk of bias assessment questions with possible answers**

| Questions | Yes | Partially | No | Unable to Determine |
| --- | --- | --- | --- | --- |
| Q1: Is the hypothesis/aim/objective of the study clearly described? | 1 | - | 0 | - |
| Q2: Are the main outcomes to be measured clearly described in the Introduction or Methods section? | 1 | - | 0 | - |
| Q3: Are the characteristics of the patients included in the study clearly described? | 1 | - | 0 | - |
| Q5: Are the distributions of principal confounders in each group of subjects to be compared clearly described? | 2 | 1 | 0 | - |
| Q6: Are the main findings of the study clearly described? | 1 | - | 0 | - |
| Q7: Does the study provide estimates of the random variability in the data for the main outcomes? | 1 | - | 0 | - |
| Q10: Have actual probability values been reported (e.g. 0.035 rather than <0.05) for the main outcomes except where the probability value is less than 0.001? | 1 | - | 0 | - |
| Q11: Were the subjects asked to participate in the study representative of the entire population from which they were recruited? | 1 | - | 0 | 0 |
| Q12: Were those subjects who were prepared to participate representative of the entire population from which they were recruited? | 1 | - | 0 | 0 |
| Q15: Was an attempt made to blind those measuring the main outcomes? | 1 | - | 0 | 0 |
| Q18: Were the statistical tests used to assess the main outcomes appropriate? | 1 | - | 0 | 0 |
| Q20: Were the main outcome measures used accurate (valid and reliable)? | 1 | - | 0 | 0 |
| Q21: Were the patients in different intervention groups (trials and cohort studies) or were the cases and controls (case-control studies) recruited from the same population? | 1 | - | 0 | 0 |
| Q22: Were study subjects in different intervention groups (trials and cohort studies) or were the cases and controls (case-control studies) recruited over the same period of time? | 1 | - | 0 | 0 |
| Q25: Was there adequate adjustment for confounding in the analyses from which the main findings were drawn? | 1 | - | 0 | 0 |

**Electronic Supplementary Material Appendix S3. Methodological quality assessment results of the excluded low quality studies**

| Authors | Title | 1 | 2 | 3 | 5 | 6 | 7 | 10 | 11 | 12 | 15 | 18 | 20 | 21 | 22 | 25 | Total |
| --- | --- | --- | --- | --- | --- | --- | --- | --- | --- | --- | --- | --- | --- | --- | --- | --- | --- |
| Blackburn, T. and Pietrosimone, B. and Goodwin, J. S. and Johnston, C. and Spang, J. T. | Co-activation during gait following anterior cruciate ligament reconstruction | 1 | 1 | 1 | 1 | 1 | 1 | 1 | 0 | 0 | 0 | 1 | 1 | 0 | 0 | 0 | 9 |
| Alkjaer, T. and Simonsen, E. B. and JÃ¸rgensen, U. and Dyhre-Poulsen, P. | Evaluation of the walking pattern in two types of patients with anterior cruciate ligament deficiency: copers and non-copers | 1 | 1 | 1 | 1 | 1 | 1 | 1 | 0 | 0 | 0 | 1 | 1 | 0 | 0 | 0 | 9 |
| Alkjaer, T. and Simonsen, E. B. and Magnusson, S. P. and Dyhre-Poulsen, P. and Aagaard, P. | Antagonist muscle moment is increased in ACL deficient subjects during maximal dynamic knee extension | 1 | 1 | 1 | 1 | 1 | 1 | 0 | 0 | 0 | 0 | 1 | 1 | 0 | 0 | 0 | 8 |
| Alkjaer, T. and Simonsen, E. B. and Peter Magnusson, S. P. and Aagaard, H. and Dyhre-Poulsen, P. | Differences in the movement pattern of a forward lunge in two types of anterior cruciate ligament deficient patients: copers and non-copers | 1 | 1 | 1 | 1 | 1 | 1 | 1 | 0 | 0 | 0 | 1 | 1 | 0 | 0 | 0 | 9 |
| Baumeister, J. and Reinecke, K. and Schubert, M. and Weiss, M. | Altered electrocortical brain activity after ACL reconstruction during force control | 1 | 1 | 1 | 1 | 1 | 1 | 1 | 0 | 0 | 0 | 1 | 1 | 0 | 0 | 0 | 9 |
| Binder-Macleod, B. I. and Buchanan, T. S. | Tibialis anterior volumes and areas in ACL-injured limbs compared with unimpaired | 1 | 1 | 1 | 1 | 1 | 1 | 1 | 0 | 0 | 0 | 1 | 1 | 0 | 0 | 0 | 9 |
| Boerboom, A. L. and Hof, A. L. and Halbertsma, J. P. and van Raaij, J. J. and Schenk, W. and Diercks, R. L. and van Horn, J. R. | Atypical hamstrings electromyographic activity as a compensatory mechanism in anterior cruciate ligament deficiency | 1 | 1 | 1 | 1 | 1 | 0 | 0 | 0 | 0 | 0 | 1 | 1 | 0 | 0 | 0 | 7 |
| Bryant, A. L. and Clark, R. A. and Pua, Y. H. | Morphology of hamstring torque-time curves following ACL injury and reconstruction: mechanisms and implications | 1 | 1 | 1 | 1 | 1 | 1 | 1 | 0 | 0 | 0 | 1 | 1 | 0 | 0 | 0 | 9 |
| Bryant, A. L. and Creaby, M. W. and Newton, R. U. and Steele, J. R. | Hamstring antagonist torque generated in vivo following ACL rupture and ACL reconstruction | 1 | 1 | 1 | 1 | 1 | 1 | 1 | 0 | 0 | 0 | 1 | 1 | 0 | 0 | 0 | 9 |
| Bryant, A. L. and Newton, R. U. and Steele, J. | Successful feed-forward strategies following ACL injury and reconstruction | 1 | 1 | 1 | 1 | 1 | 1 | 1 | 0 | 0 | 0 | 1 | 1 | 0 | 0 | 0 | 9 |
| Bulgheroni, P. and Bulgheroni, M. V. and Andrini, L. and Guffanti, P. and Castelli, C. | Walking in anterior cruciate ligament injuries | 1 | 1 | 1 | 1 | 1 | 0 | 0 | 0 | 0 | 0 | 1 | 1 | 0 | 0 | 0 | 7 |
| Bulgheroni, P. and Bulgheroni, M. V. and Andrini, L. and Guffanti, P. and Giughello, A. | Gait patterns after anterior cruciate ligament reconstruction | 1 | 1 | 1 | 1 | 1 | 0 | 0 | 0 | 0 | 0 | 1 | 1 | 0 | 0 | 0 | 7 |
| Busch, A. and Blasimann, A. and Henle, P. and Baur, H. | Neuromuscular activity during stair descent in ACL reconstructed patients: A pilot study | 1 | 1 | 1 | 1 | 1 | 1 | 1 | 0 | 0 | 0 | 1 | 1 | 0 | 0 | 0 | 9 |
| Busch, A. and Henle, P. and Boesch, L. and Blasimann, A. and Baur, H. | Neuromuscular control in patients with acute ACL injury during stair ascent â€“ A pilot study | 1 | 1 | 1 | 1 | 1 | 1 | 1 | 0 | 0 | 0 | 1 | 1 | 0 | 0 | 0 | 9 |
| Bush-Joseph, C. A. and Hurwitz, D. E. and Patel, R. R. and Bahrani, Y. and Garretson, R. and Bach, B. R., Jr. and Andriacchi, T. P. | Dynamic function after anterior cruciate ligament reconstruction with autologous patellar tendon | 1 | 1 | 1 | 1 | 1 | 1 | 1 | 0 | 0 | 0 | 1 | 1 | 0 | 0 | 0 | 9 |
| Coats-Thomas, M. S. and Mir and a, D. L. and Badger, G. J. and Fleming, B. C. | Effects of ACL reconstruction surgery on muscle activity of the lower limb during a jump-cut maneuver in males and females | 1 | 1 | 1 | 1 | 1 | 1 | 1 | 0 | 0 | 0 | 1 | 1 | 0 | 0 | 0 | 9 |
| Colne, P. and Thoumie, P. | Muscular compensation and lesion of the anterior cruciate ligament: contribution of the soleus muscle during recovery from a forward fall | 1 | 1 | 1 | 1 | 1 | 1 | 0 | 0 | 0 | 0 | 1 | 1 | 0 | 0 | 0 | 8 |
| Courtney, C. A. and Durr, R. K. and Emerson-Kavchak, A. J. and Witte, E. O. and Santos, M. J. | Heightened flexor withdrawal responses following ACL rupture are enhanced by passive tibial translation | 1 | 1 | 1 | 1 | 1 | 1 | 1 | 0 | 0 | 0 | 1 | 1 | 0 | 0 | 0 | 9 |
| Czamara, A. | Moments of muscular strength of knee joint extensors and flexors during physiotherapeutic procedures following anterior cruciate ligament reconstruction in males | 1 | 1 | 1 | 1 | 1 | 1 | 0 | 0 | 0 | 0 | 1 | 1 | 0 | 0 | 0 | 8 |
| Dingenen, B. and Janssens, L. and Luyckx, T. and Claes, S. and Bellemans, J. and Staes, F. F. | Lower extremity muscle activation onset times during the transition from double-leg stance to single-leg stance in anterior cruciate ligament injured subjects | 1 | 1 | 1 | 1 | 1 | 1 | 1 | 0 | 0 | 0 | 1 | 1 | 0 | 0 | 0 | 9 |
| Dur and , A. and Malouin, F. and Richards, C. L. and Bravo, G. | Intertrial reliability of work measurements recorded during concentric isokinetic knee extension and flexion in subjects with and without meniscal tears | 1 | 1 | 1 | 1 | 1 | 1 | 0 | 0 | 0 | 0 | 1 | 1 | 0 | 0 | 0 | 8 |
| Dur and , A. and Richards, C. L. and Malouin, F. | Strength recovery and muscle activation of the knee extensor and flexor muscles after arthroscopic meniscectomy. A pilot study | 1 | 1 | 1 | 1 | 1 | 1 | 0 | 0 | 0 | 0 | 1 | 1 | 0 | 0 | 0 | 8 |
| Dur and , A. and Richards, C. L. and Malouin, F. and Bravo, G. | Motor Recovery after Arthroscopic Partial Meniscectomy - Analyses of Gait and the Ascent and Descent of Stairs | 1 | 1 | 1 | 1 | 1 | 1 | 1 | 0 | 0 | 0 | 1 | 1 | 0 | 0 | 0 | 9 |
| Boo, M. E. and Garrison, J. C. and Hannon, J. P. and Creed, K. M. and Goto, S. and Grondin, A. N. and Bothwell, J. M. | Energy Absorption Contribution and Strength in Female Athletes at Return to Sport After Anterior Cruciate Ligament Reconstruction: Comparison With Healthy Controls | 1 | 1 | 1 | 1 | 1 | 1 | 1 | 0 | 0 | 0 | 1 | 1 | 0 | 0 | 0 | 9 |
| Giombini, A. and Menotti, F. and Laudani, L. and Piccinini, A. and Fagnani, F. and Di Cagno, A. and Macaluso, A. and Pigozzi, F. | Effect of whole body vibration frequency on neuromuscular activity in ACL-deficient and healthy males | 1 | 1 | 1 | 1 | 1 | 1 | 0 | 0 | 0 | 0 | 1 | 1 | 0 | 0 | 0 | 8 |
| Gokeler, A. and Hof, A. L. and Arnold, M. P. and Dijkstra, P. U. and Postema, K. and Otten, E. | Abnormal landing strategies after ACL reconstruction | 1 | 1 | 1 | 1 | 1 | 1 | 1 | 0 | 0 | 0 | 1 | 1 | 0 | 0 | 0 | 9 |
| Heroux, M. E. and Tremblay, F. | Corticomotor excitability associated with unilateral knee dysfunction secondary to anterior cruciate ligament injury | 1 | 1 | 1 | 1 | 1 | 1 | 1 | 0 | 0 | 0 | 1 | 1 | 0 | 0 | 0 | 9 |
| Hiemstra, L. A. and Webber, S. and MacDonald, P. B. and Kriellaars, D. J. | Knee strength deficits after hamstring tendon and patellar tendon anterior cruciate ligament reconstruction | 1 | 1 | 1 | 1 | 1 | 1 | 0 | 0 | 0 | 0 | 1 | 1 | 0 | 0 | 0 | 8 |
| Hollman, J. H. and Deusinger, R. H. and Van Dillen, L. R. and Matava, M. J. | Knee joint movements in subjects without knee pathology and subjects with injured anterior cruciate ligaments | 1 | 1 | 1 | 1 | 1 | 1 | 0 | 0 | 0 | 0 | 1 | 1 | 1 | 0 | 0 | 9 |
| Hunt, M. A. and S and erson, D. J. and Moffet, H. and Inglis, J. T. | Biomechanical changes elicited by an anterior cruciate ligament deficiency during steady rate cycling | 1 | 1 | 1 | 1 | 1 | 1 | 1 | 0 | 0 | 0 | 1 | 1 | 0 | 0 | 0 | 9 |
| Isaac, D. L. and Beard, D. J. and Price, A. J. and Rees, J. and Murray, D. W. and Dodd, C. A. | In-vivo sagittal plane knee kinematics: ACL intact, deficient and reconstructed knees | 1 | 1 | 1 | 1 | 1 | 1 | 1 | 0 | 0 | 0 | 1 | 1 | 0 | 0 | 0 | 9 |
| Kawahara, K. and Sekimoto, T. and Watanabe, S. and Yamamoto, K. and Tajima, T. and Yamaguchi, N. and Chosa, E. | Effect of genu recurvatum on the anterior cruciate ligament-deficient knee during gait | 1 | 1 | 1 | 1 | 1 | 1 | 0 | 0 | 0 | 0 | 1 | 1 | 0 | 0 | 0 | 8 |
| Kellis, E. and Karagiannidis, E. and Patsika, G. | Patellar tendon and hamstring moment-arms and cross-sectional area in patients with anterior cruciate ligament reconstruction and controls | 1 | 1 | 1 | 1 | 1 | 1 | 0 | 0 | 0 | 0 | 1 | 1 | 0 | 0 | 0 | 8 |
| Knezevic, O. M. and Mirkov, D. M. and Kadija, M. and Milovanovic, D. and Jaric, S. | Alternating Consecutive Maximum Contraction as a Test of Muscle Function in Athletes Following ACL Reconstruction | 1 | 1 | 1 | 1 | 1 | 1 | 0 | 0 | 0 | 0 | 1 | 1 | 0 | 0 | 0 | 8 |
| Knoll, Z. and Kiss, R. M. and Kocsis, L. | Gait adaptation in ACL deficient patients before and after anterior cruciate ligament reconstruction surgery | 1 | 1 | 1 | 1 | 1 | 0 | 0 | 0 | 0 | 0 | 1 | 1 | 0 | 0 | 0 | 7 |
| Knoll, Z. and Kocsis, L. and Kiss, R. M. | Gait patterns before and after anterior cruciate ligament reconstruction | 1 | 1 | 1 | 1 | 1 | 0 | 0 | 0 | 0 | 0 | 1 | 1 | 0 | 0 | 0 | 7 |
| Konishi, Y. | ACL repair might induce further abnormality of gamma loop in the intact side of the QF | 1 | 1 | 1 | 1 | 1 | 1 | 0 | 0 | 0 | 0 | 1 | 1 | 0 | 0 | 0 | 8 |
| Konishi, Y. and Aihara, Y. and Sakai, M. and Ogawa, G. and Fukubayashi, T. | Gamma loop dysfunction in the quadriceps femoris of patients who underwent anterior cruciate ligament reconstruction remains bilaterally | 1 | 1 | 1 | 1 | 1 | 1 | 0 | 0 | 0 | 0 | 1 | 1 | 0 | 0 | 0 | 8 |
| Konishi, Y. and Fukubayashi, T. | Relationship between muscle volume and muscle torque of the hamstrings after anterior cruciate ligament reconstruction | 1 | 1 | 1 | 1 | 1 | 1 | 0 | 0 | 0 | 0 | 1 | 1 | 0 | 0 | 0 | 8 |
| Konishi, Y. and Fukubayashi, T. and Takeshita, D. | Mechanism of quadriceps femoris muscle weakness in patients with anterior cruciate ligament reconstruction | 1 | 1 | 1 | 1 | 1 | 1 | 0 | 0 | 0 | 0 | 1 | 1 | 0 | 0 | 0 | 8 |
| Konishi, Y. and Fukubayashi, T. and Takeshita, D. | Possible mechanism of quadriceps femoris weakness in patients with ruptured anterior cruciate ligament | 1 | 1 | 1 | 1 | 1 | 1 | 0 | 0 | 0 | 0 | 1 | 1 | 0 | 0 | 0 | 8 |
| Konishi, Y. and Ikeda, K. and Nishino, A. and Sunaga, M. and Aihara, Y. and Fukubayashi, T. | Relationship between quadriceps femoris muscle volume and muscle torque after anterior cruciate ligament repair | 1 | 1 | 1 | 1 | 1 | 1 | 0 | 0 | 0 | 0 | 1 | 1 | 0 | 0 | 0 | 8 |
| Konishi, Y. and Oda, T. and Tsukazaki, S. and Kinugasa, R. and Fukubayashi, T. | Relationship between quadriceps femoris muscle volume and muscle torque at least 18 months after anterior cruciate ligament reconstruction | 1 | 1 | 1 | 1 | 1 | 1 | 1 | 0 | 0 | 0 | 1 | 1 | 0 | 0 | 0 | 9 |
| Konishi, Y. and Oda, T. and Tsukazaki, S. and Kinugasa, R. and Hirose, N. and Fukubayashi, T. | Relationship between quadriceps femoris muscle volume and muscle torque after anterior cruciate ligament rupture | 1 | 1 | 1 | 1 | 1 | 1 | 0 | 0 | 0 | 0 | 1 | 1 | 0 | 0 | 0 | 8 |
| Krolikowska, A. and Czamara, A. and Kentel, M. | Does Gracilis Tendon Harvest During ACL Reconstruction with a Hamstring Autograft Affect Torque of Muscles Responsible for Shin Rotation? | 1 | 1 | 1 | 1 | 1 | 1 | 1 | 0 | 0 | 0 | 1 | 1 | 0 | 0 | 0 | 9 |
| Kvist, J. | Sagittal plane knee motion in the ACL-deficient knee during body weight shift exercises on different support surfaces | 1 | 1 | 1 | 1 | 1 | 1 | 0 | 1 | 0 | 0 | 1 | 1 | 0 | 0 | 0 | 9 |
| Kvist, J. and Gillquist, J. | Sagittal plane knee translation and electromyographic activity during closed and open kinetic chain exercises in anterior cruciate ligament-deficient patients and control subjects | 1 | 1 | 1 | 2 | 1 | 0 | 0 | 0 | 0 | 0 | 1 | 1 | 0 | 0 | 0 | 8 |
| Labanca, L. and Laudani, L. and Mariani, P. P. and Macaluso, A. | Postural Adjustments Following ACL Rupture and Reconstruction: A Longitudinal Study | 1 | 1 | 1 | 2 | 1 | 1 | 0 | 0 | 0 | 0 | 1 | 1 | 0 | 0 | 0 | 9 |
| Labanca, L. and Laudani, L. and Mariani, P. P. and Macaluso, A. | Quadriceps muscle compensatory activations are delayed following anterior cruciate ligament reconstruction using hamstring tendon graft | 1 | 1 | 1 | 1 | 1 | 1 | 0 | 1 | 0 | 0 | 1 | 1 | 0 | 0 | 0 | 9 |
| Lass, P. and Kaalund, S. and leFevre, S. and Arendt-Nielsen, L. and Sinkjaer, T. and Simonsen, O. | Muscle coordination following rupture of the anterior cruciate ligament. Electromyographic studies of 14 patients | 1 | 1 | 1 | 1 | 1 | 1 | 0 | 0 | 0 | 0 | 1 | 1 | 0 | 0 | 0 | 8 |
| Lepley, L. K. and Thomas, A. C. and McLean, S. G. and Palmieri-Smith, R. M. | Fatigue's lack of effect on thigh-muscle activity in anterior cruciate ligament-reconstructed patients during a dynamic-landing task | 1 | 1 | 1 | 1 | 1 | 1 | 1 | 0 | 0 | 0 | 1 | 1 | 0 | 0 | 0 | 9 |
| Leporace, G. and Batista, L. A. and Pereira, G. R. and Zeitoune, G. and Oliveira, T. and Luciano, T. and Zeitoune, M. and Metsavaht, L. and Nadal, J. | Comparison of quadriceps and hamstrings activation ratio between healthy and anterior cruciate ligament reconstructed subjects | 1 | 1 | 1 | 1 | 1 | 1 | 1 | 0 | 0 | 0 | 1 | 1 | 0 | 0 | 0 | 9 |
| Melinska, A. and Czamara, A. and Szuba, L. and Bedzinski, R. | Biomechanical characteristics of the jump down of healthy subjects and patients with knee injuries | 1 | 1 | 1 | 1 | 1 | 1 | 1 | 0 | 0 | 0 | 1 | 1 | 0 | 0 | 0 | 9 |
| Mirkov, D. M. and Knezevic, O. M. and Maffiuletti, N. A. and Kadija, M. and Nedeljkovic, A. and Jaric, S. | Contralateral limb deficit after ACL-reconstruction: an analysis of early and late phase of rate of force development | 1 | 1 | 1 | 1 | 1 | 1 | 0 | 0 | 0 | 0 | 1 | 1 | 0 | 0 | 0 | 8 |
| Norte, G. E. and Hertel, J. N. and Saliba, S. A. and Diduch, D. R. and Hart, J. M. | Quadriceps and Patient-Reported Function in ACL-Reconstructed Patients: A Principal Component Analysis | 1 | 1 | 1 | 2 | 1 | 1 | 0 | 0 | 0 | 0 | 1 | 1 | 0 | 0 | 0 | 9 |
| Ortiz, A. and Olson, S. and Trudelle-Jackson, E. and Rosario, M. and Venegas, H. L. | Landing mechanics during side hopping and crossover hopping maneuvers in noninjured women and women with anterior cruciate ligament reconstruction | 1 | 1 | 1 | 1 | 1 | 0 | 1 | 0 | 0 | 0 | 1 | 1 | 1 | 0 | 0 | 9 |
| Osternig, L. R. and Caster, B. L. and James, C. R. | Contralateral hamstring (biceps femoris) coactivation patterns and anterior cruciate ligament dysfunction | 1 | 1 | 1 | 1 | 1 | 1 | 0 | 0 | 0 | 0 | 1 | 1 | 0 | 0 | 0 | 8 |
| Otzel, D. M. and Chow, J. W. and Tillman, M. D. | Long-term deficits in quadriceps strength and activation following anterior cruciate ligament reconstruction | 1 | 1 | 1 | 1 | 1 | 1 | 1 | 0 | 0 | 0 | 1 | 1 | 0 | 0 | 0 | 9 |
| Patel, R. R. and Hurwitz, D. E. and Bush-Joseph, C. A. and Bach, B. R., Jr. and Andriacchi, T. P. | Comparison of clinical and dynamic knee function in patients with anterior cruciate ligament deficiency | 1 | 1 | 1 | 1 | 1 | 1 | 1 | 0 | 0 | 0 | 1 | 1 | 0 | 0 | 0 | 9 |
| Pelegrinelli, A. R. M. and Guenka, L. C. and Dias, J. M. and Dela Bela, L. F. and Silva, M. F. and Moura, F. A. and Brown, L. E. and Cardoso, J. R. | Isokinetic Muscle Performance after Anterior Cruciate Ligament Reconstruction: A Case-Control Study | 1 | 1 | 1 | 1 | 1 | 1 | 1 | 0 | 0 | 0 | 1 | 1 | 0 | 0 | 0 | 9 |
| Pereira, H. M. and Nowotny, A. H. and Santos, A. B. and Cardoso, J. R. | Electromyographic activity of knee stabilizer muscles during six different balance board stimuli after anterior cruciate ligament surgery | 1 | 1 | 1 | 1 | 1 | 1 | 1 | 0 | 0 | 0 | 1 | 1 | 0 | 0 | 0 | 9 |
| Perry, B. D. and Levinger, P. and Morris, H. G. and Petersen, A. C. and Garnham, A. P. and Levinger, I. and McKenna, M. J. | The effects of knee injury on skeletal muscle function, Na+, K+-ATPase content, and isoform abundance | 1 | 1 | 1 | 1 | 1 | 1 | 1 | 0 | 0 | 0 | 1 | 1 | 0 | 0 | 0 | 9 |
| Pfeifer, K. and Banzer, W. | Motor performance in different dynamic tests in knee rehabilitation | 1 | 1 | 1 | 1 | 1 | 1 | 0 | 0 | 0 | 0 | 1 | 1 | 0 | 0 | 0 | 8 |
| Rebel, M. and Paessler, H. H. | The effect of knee brace on coordination and neuronal leg muscle control: an early postoperative functional study in anterior cruciate ligament reconstructed patients | 1 | 1 | 1 | 1 | 1 | 1 | 0 | 0 | 0 | 0 | 1 | 1 | 0 | 0 | 0 | 8 |
| Robbins, S. M. and Morelli, M. and Martineau, P. A. and St-Onge, N. and Boily, M. and Dimentberg, R. and Antoniou, J. | A comparison of muscle activation and knee mechanics during gait between patients with non-traumatic and post-traumatic knee osteoarthritis | 1 | 1 | 1 | 1 | 1 | 0 | 1 | 0 | 0 | 0 | 1 | 1 | 1 | 0 | 0 | 9 |
| Rudolph, K. S. and Axe, M. J. and Snyder-Mackler, L. | Dynamic stability after ACL injury: who can hop? | 1 | 1 | 1 | 1 | 1 | 1 | 1 | 0 | 0 | 0 | 1 | 1 | 0 | 0 | 0 | 9 |
| Serrancoli, G. and Monllau, J. C. and Font-Llagunes, J. M. | Analysis of muscle synergies and activation-deactivation patterns in subjects with anterior cruciate ligament deficiency during walking | 1 | 1 | 1 | 1 | 1 | 1 | 0 | 0 | 0 | 0 | 1 | 1 | 0 | 0 | 0 | 8 |
| Sinkjaer, T. and Arendtnielsen, L. | Knee Stability and Muscle Coordination in Patients with Anterior Cruciate Ligament Injuries - an Electromyographic Approach | 1 | 1 | 1 | 1 | 1 | 1 | 0 | 0 | 0 | 0 | 1 | 1 | 0 | 0 | 0 | 8 |
| Steele, J. R. and Brown, J. M. | Effects of chronic anterior cruciate ligament deficiency on muscle activation patterns during an abrupt deceleration task | 1 | 1 | 1 | 1 | 1 | 1 | 1 | 0 | 0 | 0 | 1 | 1 | 0 | 0 | 0 | 9 |
| Sturnieks, D. L. and Besier, T. F. and Lloyd, D. G. | Muscle activations to stabilize the knee following arthroscopic partial meniscectomy | 1 | 1 | 1 | 1 | 1 | 1 | 1 | 0 | 0 | 0 | 1 | 1 | 0 | 0 | 0 | 9 |
| Suarez, T. and Laudani, L. and Giombini, A. and Saraceni, V. M. and Mariani, P. P. and Pigozzi, F. and Macaluso, A. | Comparison in Joint-Position Sense and Muscle Coactivation Between Anterior Cruciate Ligament-Deficient and Healthy Individuals | 1 | 1 | 1 | 1 | 1 | 1 | 0 | 0 | 0 | 0 | 1 | 1 | 0 | 0 | 0 | 8 |
| Swanik, C. B. and Lephart, S. M. and Swanik, K. A. and Stone, D. A. and Fu, F. H. | Neuromuscular dynamic restraint in women with anterior cruciate ligament injuries | 1 | 1 | 1 | 1 | 1 | 1 | 1 | 0 | 0 | 0 | 1 | 1 | 0 | 0 | 0 | 9 |
| Teixeira da Fonseca, S. and Silva, P. L. and Ocarino, J. M. and Guimaraes, R. B. and Oliveira, M. T. and Lage, C. A. | Analyses of dynamic co-contraction level in individuals with anterior cruciate ligament injury | 1 | 1 | 1 | 1 | 1 | 1 | 1 | 0 | 0 | 0 | 1 | 1 | 0 | 0 | 0 | 9 |
| Torry, M. R. and Decker, M. J. and Ellis, H. B. and Shelburne, K. B. and Sterett, W. I. and Steadman, J. R. | Mechanisms of compensating for anterior cruciate ligament deficiency during gait | 1 | 1 | 1 | 1 | 1 | 1 | 0 | 0 | 0 | 0 | 1 | 1 | 1 | 0 | 0 | 9 |
| Urbach, D. and Awiszus, F. | Impaired ability of voluntary quadriceps activation bilaterally interferes with function testing after knee injuries. A twitch interpolation study | 1 | 1 | 1 | 1 | 1 | 1 | 1 | 0 | 0 | 0 | 1 | 1 | 0 | 0 | 0 | 9 |
| Urbach, D. and Nebelung, W. and Becker, R. and Awiszus, F. | Effects of reconstruction of the anterior cruciate ligament on voluntary activation of quadriceps femoris - A prospective twitch interpolation study | 1 | 1 | 1 | 1 | 1 | 1 | 1 | 0 | 0 | 0 | 1 | 1 | 0 | 0 | 0 | 9 |
| Urbach, D. and Nebelung, W. and Weiler, H. T. and Awiszus, F. | Bilateral deficit of voluntary quadriceps muscle activation after unilateral ACL tear | 1 | 1 | 1 | 1 | 1 | 1 | 1 | 0 | 0 | 0 | 1 | 1 | 0 | 0 | 0 | 9 |
| Vairo, G. L. and Myers, J. B. and Sell, T. C. and Fu, F. H. and Harner, C. D. and Lephart, S. M. | Neuromuscular and biomechanical landing performance subsequent to ipsilateral semitendinosus and gracilis autograft anterior cruciate ligament reconstruction | 1 | 1 | 1 | 1 | 1 | 1 | 1 | 0 | 0 | 0 | 1 | 1 | 0 | 0 | 0 | 9 |
| Vanlent, M. E. T. and Drost, M. R. and V and erwildenberg, Fajm | Emg Profiles of Acl-Deficient Patients during Walking - the Influence of Mild Fatigue | 1 | 1 | 1 | 1 | 1 | 1 | 1 | 0 | 0 | 0 | 1 | 1 | 0 | 0 | 0 | 9 |
| Williams, G. N. and Barrance, P. J. and Snyder-Mackler, L. and Axe, M. J. and Buchanan, T. S. | Specificity of muscle action after anterior cruciate ligament injury | 1 | 1 | 1 | 1 | 1 | 1 | 1 | 0 | 0 | 0 | 1 | 1 | 0 | 0 | 0 | 9 |
| Williams, G. N. and Barrance, P. J. and Snyder-Mackler, L. and Buchanan, T. S. | Altered quadriceps control in people with anterior cruciate ligament deficiency | 1 | 1 | 1 | 1 | 1 | 1 | 1 | 0 | 0 | 0 | 1 | 1 | 0 | 0 | 0 | 9 |
| Wojtys, E. M. and Huston, L. J. | Neuromuscular Performance in Normal and Anterior Cruciate Ligament-Deficient Lower-Extremities | 1 | 1 | 1 | 1 | 1 | 0 | 0 | 1 | 0 | 0 | 1 | 1 | 0 | 0 | 0 | 8 |
| Zhang, L. Q. and Nuber, G. W. and Bowen, M. K. and Koh, J. L. and Butler, J. P. | Multiaxis muscle strength in ACL deficient and reconstructed knees: compensatory mechanism | 1 | 1 | 1 | 1 | 1 | 1 | 1 | 0 | 0 | 0 | 1 | 1 | 0 | 0 | 0 | 9 |
| Huber, J.; Lisinski, P.; Kloskowska, P.; Gronek, A.; Lisiewicz, E.; Trzeciak, T.; | Meniscus suture provides better clinical and biomechanical results at 1-year follow-up than meniscectomy | 1 | 1 | 1 | 1 | 1 | 0 | 0 | 0 | 0 | 0 | 1 | 1 | 1 | 1 | 0 | 9 |

**Electronic Supplementary Material Appendix S4. Characteristics of included studies**

| **Author/Year**  **(Study Design)** | **Sample Size**  **(Males/Females)** | **Age**  **(Mean(SD) or**  **Mean(Range)** | **Injury Type** | **Surgery Type** | **Time of Assessment**  **Post-injury** | **Time of Assessment**  **Post-surgery** | **Outcome measures** |
| --- | --- | --- | --- | --- | --- | --- | --- |
| Anterior cruciate ligament (ACL) injuries | | | | | | | |
| Almeida et al. 2018 [41] Prospective Case-Control | Injured: n=20 (20/0)  Control: n=20 (20/0) | Injured: 21 (18-28)  Control: 20.5 (18-34) | ACL | ACLR (4-stranded ST+gracilis) | 3 (1-12) Months  (Pre-op) | Pre-op  6 Months | Quadriceps and hamstring concentric strength |
| Chung et al. 2015 [42]  Prospective Case-Control | Injured: n=76 (65/11)  Control: n=76 (65/11) | Injured: 27.9 (8.6)  Control: 27.6 (7) | ACL | ACLR (ST) | NR | 3-6-12-24 Months | Quadriceps and hamstring concentric strength |
| Clagg et al. 2015 [43]  Cross-Sectional | Injured: n=66 (20/46)  Control: n=47 (15/32) | Injured: 17.6 (2.9)  Control: 17.0 (2.3) | ACL | ACLR (BPTB(26),hamstring(32), allograft(8) | NR | 6.7 (1.6) Months | Quadriceps and hamstring concentric strength |
| Engelen-van Melick et al. 2017 [44]  Cross-Sectional | Injured: n=97 (51/46)  Control: n=44 (22/22) | Injured:  Male BPTB: 36.9 (8.9)  Male STG: 37.6 (9.8)  Female BPTB: 36.0 (11.0)  Female STG: 33.5 (10.0)  Control:  Male: 35.3 (10.6)  Female: 31.5 (11.9) | ACL | ACLR (BPTB(47), STG(50)) | NR | 54 (22.8) Months | Quadriceps and hamstring concentric and eccentric strength |
| Freddolini et al. 2015 [45]  Cross-Sectional | Injured: n=15 (15/0)  Control: n=15 (15/0) | Injured: 30.4 (6.1)  Control: 30.2 (7.4) | ACL | ACLR  (ST) | NR | 2 Years | Electromechanical delay (BF, ST) |
| Garrison et al. 2018 [46]  Cross-Sectional | Injured: n=24  Control: n=24 | Injured: 15.5 (1.3)  Control: 15.5 (1.2) | ACL | ACLR  (NR) | NR | 3 Months | Quadriceps concentric strength |
| Goetschius and Hart 2016 [47]  Cross-Sectional | Injured: n=53 (27/26)  Control: n=50 (28/22) | Injured: 23.4 (4.9)  Control: 23.3 (4.4) | ACL | ACLR  (NR) | NR | 44.1 (29.9) Months | Quadriceps isometric strength, voluntary activation and torque variability |
| Goetschius et al. 2019 [48]  Non-Randomized Intervention | Injured: n=51 (14/37)  Control: n=21 (8/13) | Injured:  BPTB: 24.1 (5.4)  Non-BPTB: 21.7 (3.4)  Control: 22.2 (3.1) | ACL | ACLR (BPTB(25), hamstring(17), allograft(8)) | NR | BPTB: 4.7 (3.2) Years  Non-BPTB: 4.2 (3.8) Years | Quadriceps isometric strength |
| Harkey et al. 2016 [10]  Cross-Sectional | Injured: n=73 (24/49)  Control: n=74 (24/50) | Injured: 21.4 (3.7)  Control: 21.4 (2.6) | ACL | ACLR (PT(40), STG(30), allograft(3)) | NR | 39.6 (38.7) Months | Quadriceps H-reflex, voluntary activation |
| Holsgaard-Larsen et al. 2014 [50]  Cross-Sectional | Injured: n=23 (23/0)  Control: n=25 (25/0) | Injured: 27.2 (7.5)  Control: 27.2 (5.4) | ACL | ACLR (Hamstring) | NR | 26.5 (6.6) Months | Quadriceps and hamstring isometric strength |
| Hsiao et al. 2014 [51]  Prospective Observational | Injured: n=12 (9/3)  Control: n= 15 (11/4) | Injured: 25.7 (9.3)  Control: 23.0 (3.3) | ACL | ACLR (BPTB) | 18.5 (22.7) Months (Pre-op) | Pre-op  3 and 6 Months | Quadriceps and hamstring isometric and concentric strength |
| Hsieh et al. 2015 [52]  Cross-Sectional | Injured: n=28 (14/14)  Control: n=28 (14/14) | Injured: 19.6 (4.5)  Control: 20.0 (4.3) | ACL | ACLR (Hamstring(16), PT(1), allograft(11) | NR | 86.2 (79-96) Days | Quadriceps concentric strength, rate of torque development, time to peak torque |
| Johnson et al. 2018 [54]  Cross-Sectional | Injured: n=67 (43/24)  Control: n=10 (6/4) | Injured: 21.34 (5.73)  Control: 23.50 (3.44) | ACL | ACLR (PT) | NR | 7.52 (1.36) Months | Quadriceps concentric and isometric strength, rate of torque development, time to peak torque, voluntary activation |
| Kaminska et al. 2015 [55]  Cross-Sectional | Injured:  ACL: n=10 (10/0)  ACL+PCL: n=9 (9/0)  Control: n=12 (12/0) | Injured:  ACL: 27.1 (7.4)  ACL+PCL: 30.1 (10.6)  Control: 29.8 (9.0) | ACL  ACL+PCL | n/a | ACL: 7 (1-16) Months  ACL+PCL: 8.5 (4-12) Months | n/a | Quadriceps and hamstring concentric strength |
| Kellis et al. 2019 [56]  Cross-Sectional | Injured: n=14 (0/34)  Control: n=34 (0/34) | Injured: 21.9 (2.1)  Control: 21.5 (2.5) | ACL | ACLR (STG) | NR | 7-12 Months | Quadriceps and hamstring concentric and eccentric strength |
| Kline et al. 2018 [57]  Cross-Sectional | Injured: n=20 (9/11)  Control: n=45 (23/22) | Injured: 22.8 (15-45)  Control: 25.8 (15-45) | ACL | ACLR (BPTB(12), hamstring(8) | NR | 8.3 (2) Months | Quadriceps isometric strength |
| Krishnan and Williams 2011 [58]  Cross-Sectional | Injured: n=15  Control: n=15 | Injured: 24.73 (4.98)  Control: 24.73 (3.71) | ACL | ACLR (NR) | NR | 2-15 Years | Quadriceps and hamstring isometric strength, voluntary activation |
| Kuenze et al. 2015 [20]  Cross-Sectional | Injured: n=22 (12/10)  Control: n=24 (12/12) | Injured: 22.5 (5)  Control: 21.7 (3.6) | ACL | ACLR (Hamstring(12), BPTB(10)) | NR | 31.5 (23.5) (7-80) Months | Quadriceps isometric strength, voluntary activation, H-reflex, active motor threshold |
| Kuenze et al. 2017 [59]  Non-Randomized Intervention | Injured: n=10 (1/9)  Control: n=10 (1/9) | Injured: 21 (2.8)  Control: 20.8 (2.5) | ACL | ACLR (ST(5), BPTB(5)) | NR | 27.9 (16.6) Months | Quadriceps isometric strength, voluntary activation |
| Kvist et al. 2001 [60]  Cross-Sectional | Injured: n=12 (8/4)  Control: n=11 (5/6) | Injured: 29 (14-37)  Control: 27 (19-34) | ACL | n/a | 19 (3) Months | n/a | Quadriceps concentric and eccentric strength |
| Larsen et al. 2015 [11]  Cross-Sectional | Injured: n=16 (9/7)  Control: n=16 (9/7) | Injured: 29.6 (9.7)  Control: 29.9 (9) | ACL | ACLR  (BPTB(2), STG(14)) | NR | 11.1 (0.8) Months | Quadriceps and hamstring isometric, concentric and eccentric strength, rate of torque development |
| Lepley et al. 2014 [61]  Cross-Sectional | Injured: n=29 (9/20)  Control: n=29 (9/20) | Injured: 21.2 (3.7)  Control: 21.5 (2.7) | ACL | ACLR (PT(12), hamstring(14), allograft(3)) | NR | 48.2 (35.5) Months | Quadriceps isometric strength, voluntary activation, H-reflex, active motor threshold |
| Lepley et al. 2015 [9]  Prospective Observational | Injured: n=20 (9/11)  Control: n=20 (9/11) | Injured: 20.9 (4.4)  Control: 21.7 (3.7) | ACL | ACLR (BPTB(11), hamstring(9)) | 37.1 (15.3) Days  (Pre-op) | Pre-op  15.9 (2.4) Days  28.3 (2.9) Weeks | Quadriceps isometric strength, voluntary activation, H-reflex, active motor threshold, motor-evoked potentials |
| Lepley et al. 2019 [62]  Cross-Sectional | Injured: n=11 (5/6)  Control: n=11 (5/6) | Injured: 22.6 (1.8)  Control: 23.2 (1.6) | ACL | ACLR (PT(9), STG(2)) | NR | 69.4 (22.4) Months | Quadriceps isometric strength, H-reflex, active motor threshold, motor-evoked potentials, muscle volume |
| Maeda et al. 2018 [63]  Cross-Sectional | Injured: n=10 (3/7)  Control: n=10 (3/7) | Injured: 23.9 (4.4)  Control: 21.6 (3.1) | ACL | ACLR (Hamstring(9), BPTB(1)) | NR | At Least 24 Months | Quadriceps and hamstring concentric strength |
| Norte et al. 2018 [64]  Cross-Sectional | Injured:  Early: n=34 (20/14)  Late: n=30 (10/20)  Control: n=30 (12/18) | Injured:  Early: 22.5 (6.3)  Late: 24.9 (5.9)  Control: 22.7 (4.6) | ACL | ACLR (PT(26), hamstring(29), allograft(9)) | NR | Early: 9 (4.3) Months  Late: 70.5 (41.6) Months | Quadriceps isometric strength, voluntary activation, H-reflex, active motor threshold |
| Oeffinger et al. 2001 [65]  Cross-Sectional | Injured: n=15 (15/0)  Control: n=8 (8/0) | Injured:  Allograft: 25.4 (4)  Autograft: 27 (5)  Control: 26.2 (3) | ACL | ACLR (BPTB(8), allograft(7)) | NR | Allograft: 21.3 (5) Months  Autograft: 27.6 (10) Months | Quadriceps and hamstring concentric strength |
| O'Malley et al. 2018 [66]  Cross-Sectional | Injured: n=118 (118/0)  Control: n=44 (44/0) | Injured: 23.6 (5.8)  Control: 24.1 (3.6) | ACL | ACLR (PT) | NR | 6.6 (1.0) Months | Quadriceps and hamstring concentric strength |
| Pamukoff et al. 2017 [22]  Cross-Sectional | Injured: n=20 (6/14)  Control: n=20 (6/14) | Injured: 21.1 (1.7)  Control: 21.2 (1.1) | ACL | ACLR (PT(16), hamstring(3), allograft(1)) | NR | 50.7 (21.3) Months | Quadriceps isometric strength, rate of torque development, voluntary activation |
| Pamukoff et al. 2018 [67]  Cross-Sectional | Injured: n=38 (9/29)  Control: n=38 (9/29) | Injured: 21.9 (2.4)  Control: 21.9 (1.3) | ACL | ACLR (PT(21), hamstring(9), allograft(8)) | NR | 48.0 (25.0) Months | Quadriceps isometric and concentric strength, rate of torque development |
| Reed-Jones and Vallis 2008 [68]  Cross-sectional | Injured: n=8 (4/4)  Control: n=8 (4/4) | Injured: 31.3 (8.9)  Control: 29.5 (8.7) | ACL | n/a | 6 (7.4) (0.25-19) Years | n/a | Quadriceps and hamstring concentric strength |
| Ristanis et al. 2009 [69]  Cross-Sectional | Injured: n=12 (12/0)  Control: n=12 (12/0) | Injured: 26 (8)  Control: 29 (5) | ACL | ACLR (STG) | NR | 24-26 Months | Electromechanical delay (BF, ST) |
| Roos et al. 2014 [70]  Cross-Sectional | Injured: ACLD: n=20 (17/3)  ACLR: n=21 (16/5)  Control: n=21 (12/9) | Injured: ACLD: 29 (6)  ACLR: 29 (9)  Control: 27(8) | ACL | ACLD and ACLR (STG) | ACLD: 19 (52) Months | ACLR: 13 (9) Months | Quadriceps and hamstring concentric strength |
| Scheurer et al. 2020 [71]  Cross-Sectional | Injured: n=16 (8/8)  Control: n=16 (8/8) | Injured: 20.4 (1.8)  Control: 21.0 (1.7) | ACL | ACLR (PT(4), HT(9), allograft(2), repair(1)) | NR | 33.9 (26.1) Months | Quadriceps isometric strength, voluntary activation, active motor threshold, electromechanical delay, rate of torque development, torque variability |
| Tengman et al. 2014 [12]  Non-Randomized  Intervention | Injured: ACLR: n=33 (21/12)  ACLD: n=37 (23/14)  Control: n=33 (21/12) | Injured: ACLR: 45.6 (4.5)  ACLD: 48.1 (5.9)  Control: 46.7 (5.0) | ACL | ACLR (Patellar (19), Aiming device (9), BPTB (5)) and ACLD | ACLR=23.9 (2.8) Years  ACLD=23.1 (1.3) Years | NR | Quadriceps and hamstring concentric and eccentric strength |
| Thomas et al. 2015 [73]  Cross-Sectional | Injured: n=17 (10/7)  Control: n=16 (5/11) | Injured: 21.41 (4.73)  Control: 23.38 (4.11) | ACL | ACLR (PT(10), HT(7) | NR | 7-10 Months | Quadriceps isometric strength, voluntary activation |
| Tourville et al. 2014 [76]  Prospective Observational | Injured: n=39 (19/20)  Control: n=32 (14/18) | Injured: 28 (12)  Control: 27 (7) | ACL | ACLR (BPTB(35), HT(1), allograft(3)) | NR | 3 Weeks  1 Year  46 (9.5) Months | Quadriceps and hamstring concentric strength |
| Tsarouhas et al. 2015 [77]  Cross-Sectional | Injured: Single bundle: n=15 (15/0)  Double bundle: n=15 (15/0)  Control: n=15 (15/0) | Injured: Single bundle: 24.1 (4.9)  Double bundle: 26 (4.6)  Control: 25.2 (1.3) | ACL | ACLR (HT) | NR | Single bundle: 19 (5.3) Months  Double bundle: 17.2 (4.9) Months | Quadriceps and hamstring concentric strength |
| Vairo 2014 [78]  Cross-Sectional | Injured: n=15 (0/15)  Control: n=15 (0/15) | Injured: 20.47 (1.96)  Control: 20.93 (1.22) | ACL | ACLR (STG) | NR | 25.93 (11.25) Months | Hamstring concentric strength |
| Vairo et al. 2013 [79]  Cross-Sectional | Injured: n=15 (0/15)  Control: n=15 (0/15) | Injured: 20.47 (1.96)  Control: 20.93 (1.22) | ACL | ACLR (STG) | NR | 25.93 (11.25) Months | Quadriceps and hamstring concentric strength |
| Ward et al. 2019 [80]  Cross-Sectional | Injured: n=18 (12/6)  Control: n=18 (12/6) | Injured: 29.6 (8.4)  Control: 29.2 (6.8) | ACL | n/a | 69.6 (42.5) (11-138) Days | n/a | Quadriceps torque variability, active motor threshold |
| Welling et al. 2019 [81]  Prospective Observational | Injured: n=38 (38/0)  Control: n=30 (30/0) | Injured: 24.2 (4.7)  Control: 22.8 (2.5) | ACL | ACLR (HT(24), BPTB(14)) | NR | 3.9 (1.1),  6.6 (0.7),  9.7 (0.8) Months | Quadriceps and hamstring concentric strength |
| Xergia et al. 2013 [82]  Cross-Sectional | Injured: n=22 (22/0)  Control: n=22 (22/0) | Injured: 28.8 (11.2)  Control: 24.8 (9.1) | ACL | ACLR (BPTB) | NR | 7.0 (0.9) Months | Quadriceps and hamstring concentric strength |
| Zarzycki et al. 2018 [83]  Cross-Sectional | Injured: n=18 (8/10)  Control: n=18 (8/10) | Injured: 21.8 (3.3)  Control: 22.2 (2.5) | ACL | ACLR (BPTB(5), HT(8), allograft(3)) | NR | 2 Weeks | Quadriceps H-reflex, motor evoked potentials |
| Zult et al. 2017 [84]  Cross-Sectional | Injured: n=32 (16/16)  Control: n=40 (20/20) | Injured: 23 (4)  Control: 22 (1.5) | ACL | n/a | Less Than 2 Years  208 (145) (60-664) Days | n/a | Quadriceps and hamstring isometric, concentric and eccentric strength, voluntary activation, torque variability |
| Zwolski et al. 2016 [85]  Cross-Sectional | Injured:  Unilateral: n=16 (0/16)  Bilateral: n=16 (0/16)  Control: n=16 (0/16) | Injured: Unilateral: 18.2 (2.6)  Bilateral: 18.9 (3.6)  Control: 17.9 (2.2) | ACL | ACLR (NR) | NR | Unilateral: 9.2 (2.2) Months  Bilateral: 10.6 (7.6) Months | Quadriceps isometric strength |
| Meniscus injuries | | | | | | | |
| Hall et al. 2013 [49]  Prospective Observational | Injured: n=82 (72/10)  Control: n=38 (32/6) | Injured: 41.0 (5.4)  Control: 40.8 (6.5) | Meniscus | Meniscectomy | NR | 3 Months  2.3 Years (2.4 Months) | Quadriceps and hamstring concentric and eccentric strength |
| Ilich et al. 2013 [53]  Cross-Sectional | Injured: n=21 (8/13)  Control: n=21 (8/13) | Injured: 45.7 (6.1)  Control: 43.6 (5.7) | Meniscus | Meniscectomy | NR | 11 (6) Weeks | Quadriceps concentric strength |
| Sturnieks et al. 2008 [72]  Cross-Sectional | Injured: n=102 (87/15)  Control: n=42 (25/17) | Injured: 39.7 (7.4)  Control: 37.6 (7.5) | Meniscus | Meniscectomy | NR | 11(4.3) Weeks | Quadriceps and hamstring isometric and concentric strength |
| Thorlund et al. 2010 [74]  Cross-Sectional | Injured: n=31 (21/10)  Control: n=31 (19/12) | Injured: 46.0 (5.5)  Control: 45.9 (5.8) | Meniscus | Meniscectomy | NR | 20.6 (6.1) Months | Quadriceps and hamstring isometric, concentric and eccentric strength, rate of torque development |
| Thorlund et al. 2012 [75]  Prospective Observational | Injured: n=22 (17/5)  Control: n=25 (14/11) | Injured: 46.6 (5.0)  Control: 46.4 (5.2) | Meniscus | Meniscectomy | NR | 21.6 (5.1) Months  49.6 (5.0) Months | Quadriceps and hamstring isometric strength, rate of torque development |
| ACLR anterior cruciate ligament reconstruction, ACLD anterior cruciate ligament deficient, ST semitendinosus, BPTB bone patellar tendon bone, STG semitendinosus graft, NR not reported, PT patellar tendon, HT hamstring tendon | | | | | | | |

**Electronic Supplementary Material Appendix S5. Forest plots and funnel plots**

1. **ACL Studies**
   1. Quadriceps muscle size


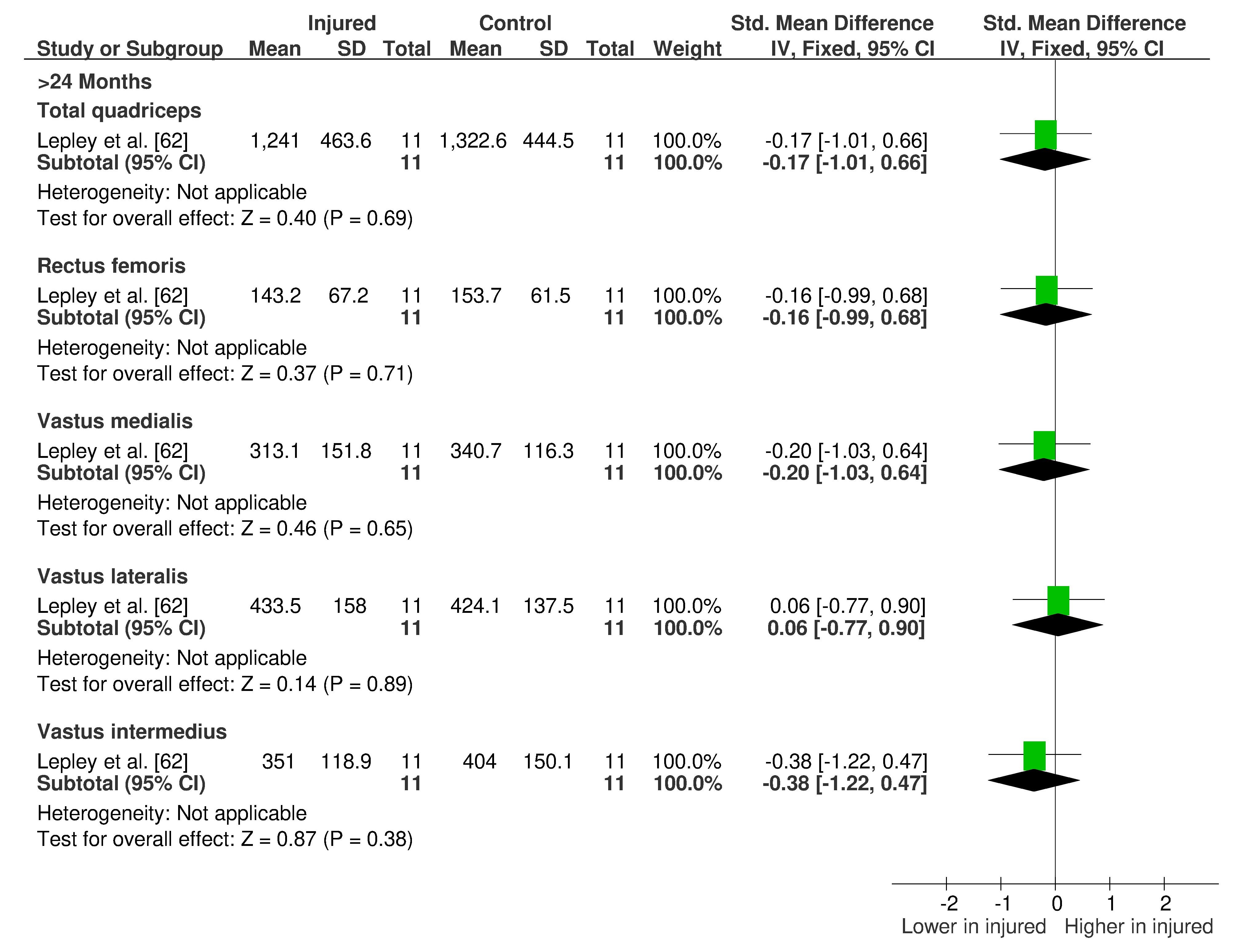


- 1. Quadriceps isometric strength


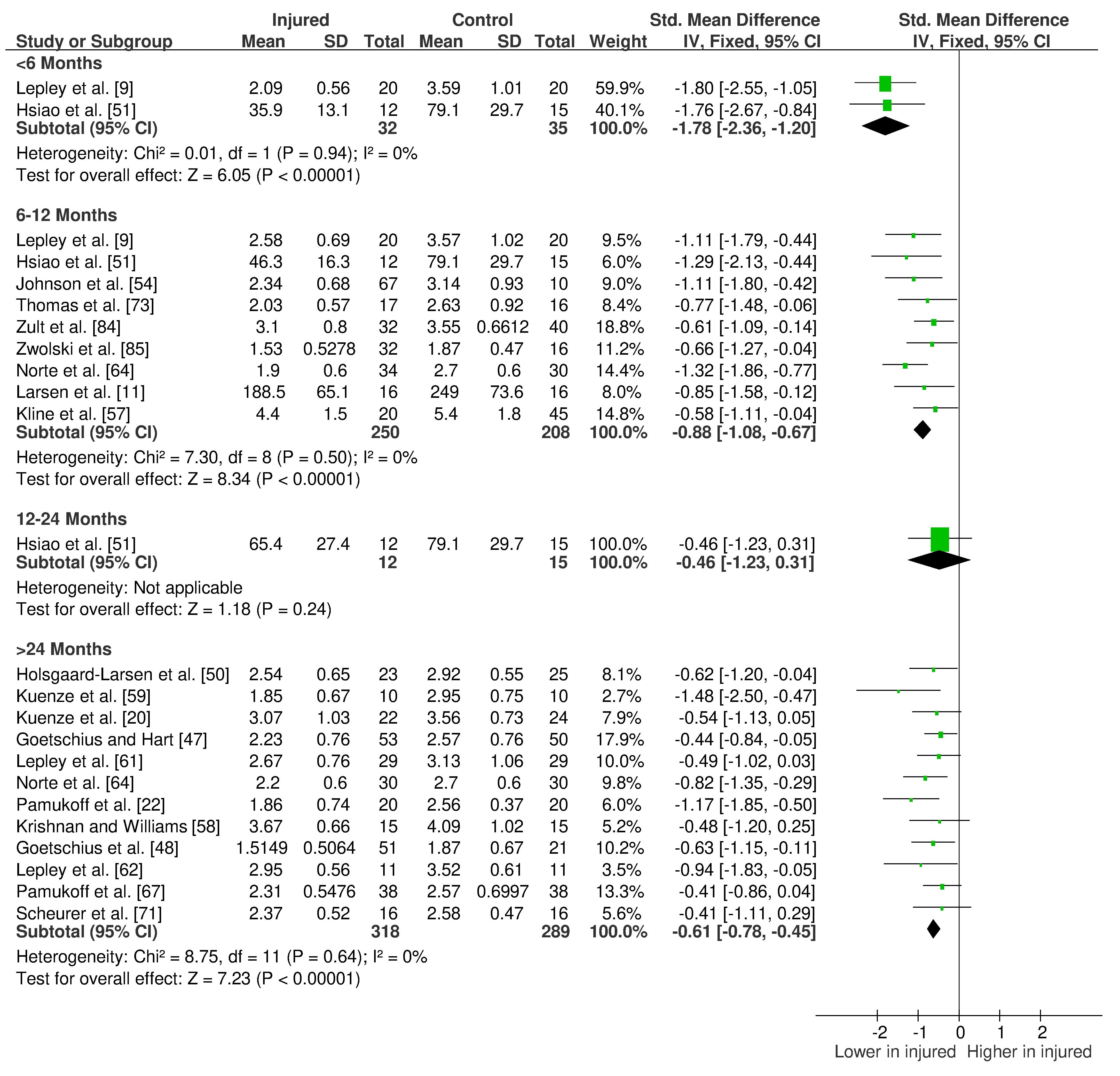


- 1. Funnel plot for quadriceps isometric strength


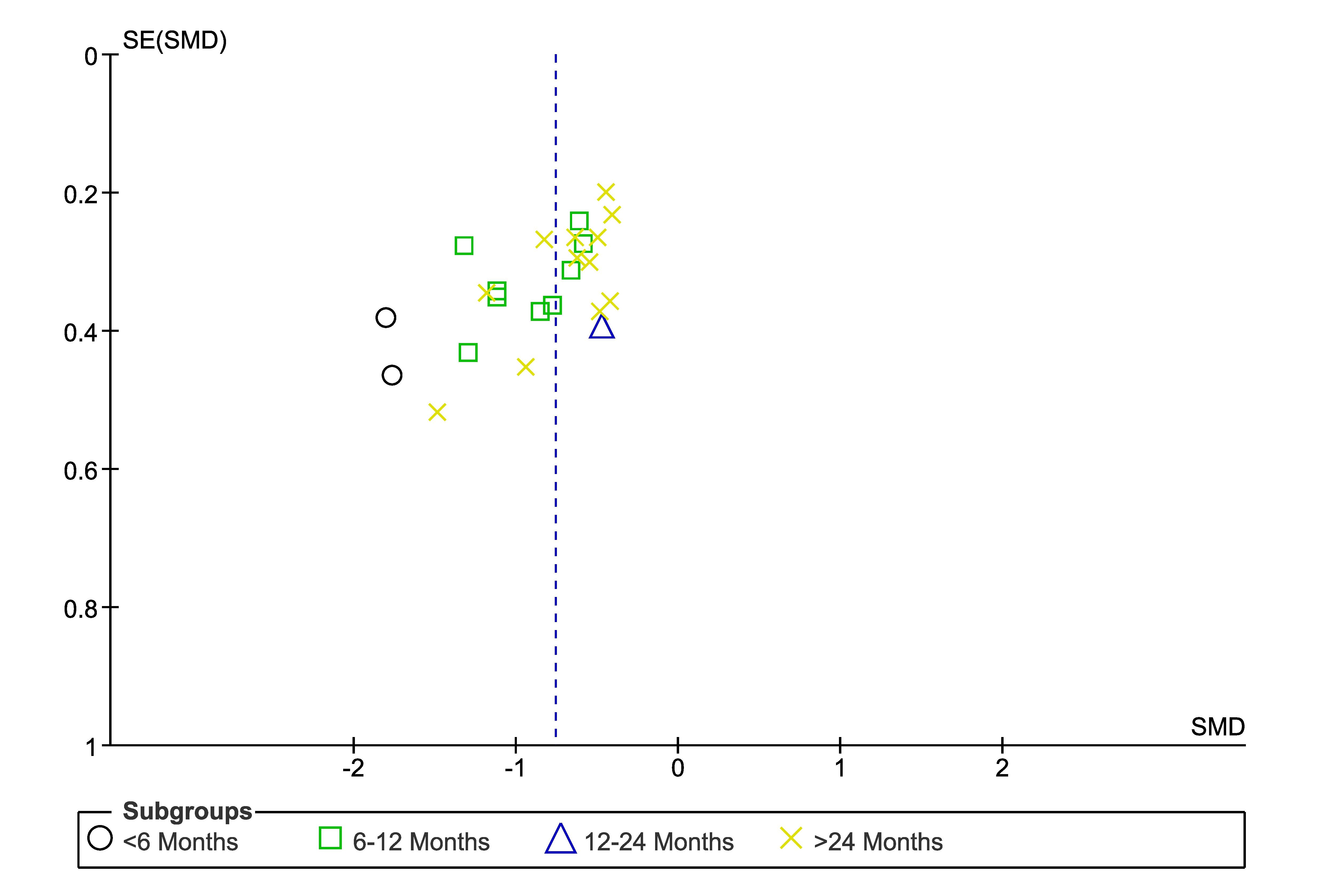


- 1. Quadriceps fast concentric strength


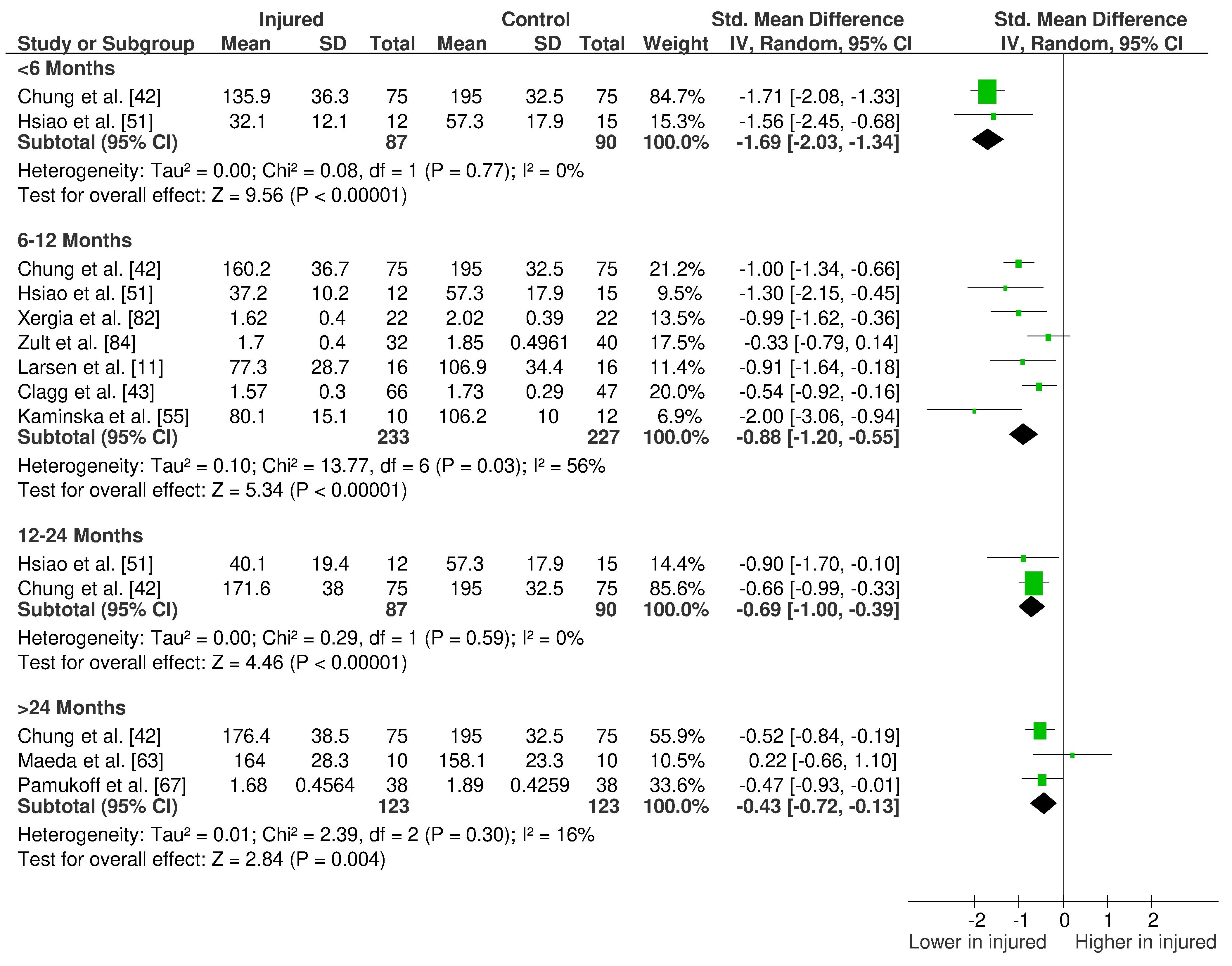


- 1. Quadriceps eccentric strength


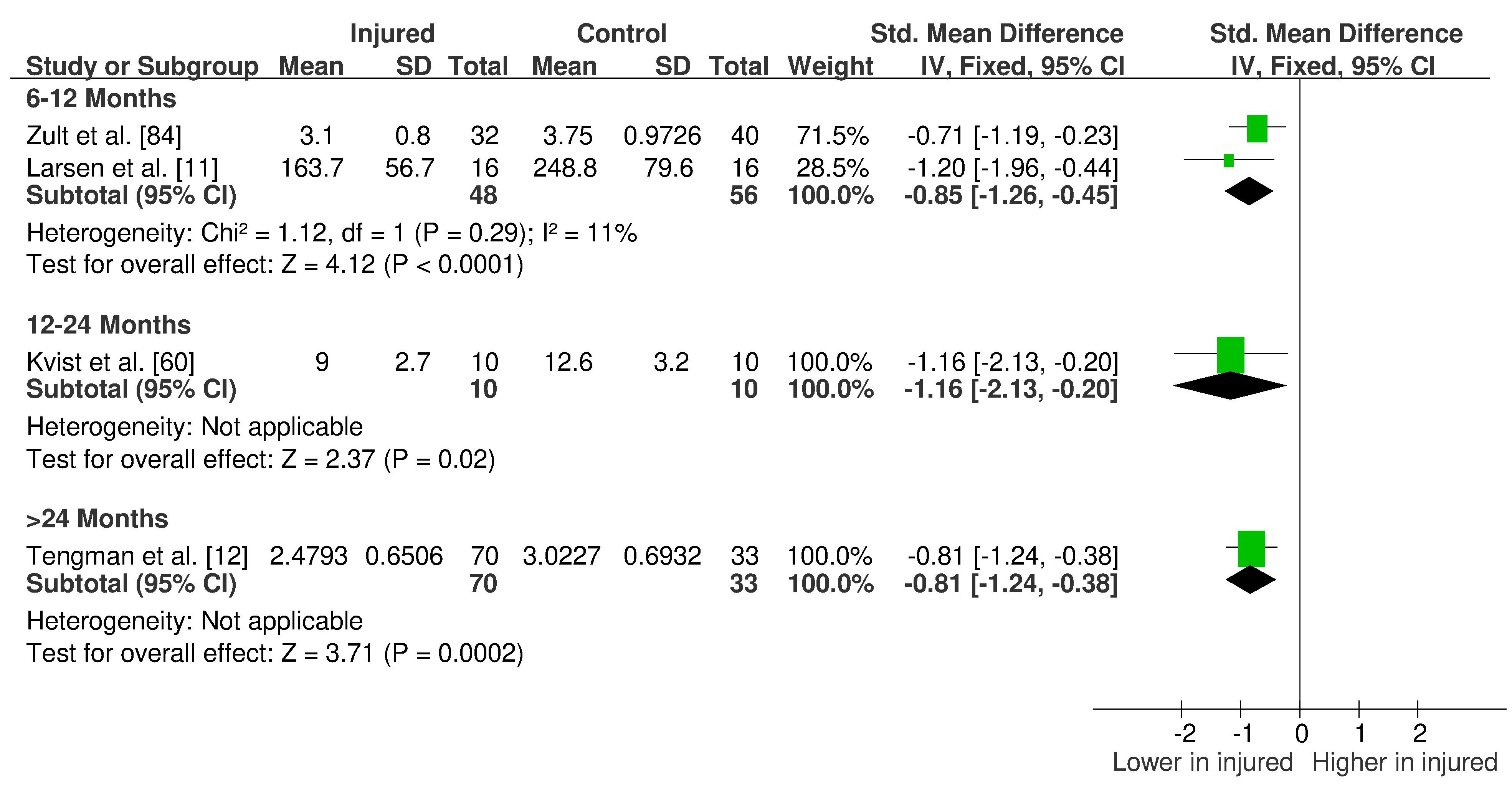


- 1. Quadriceps rate of torque development


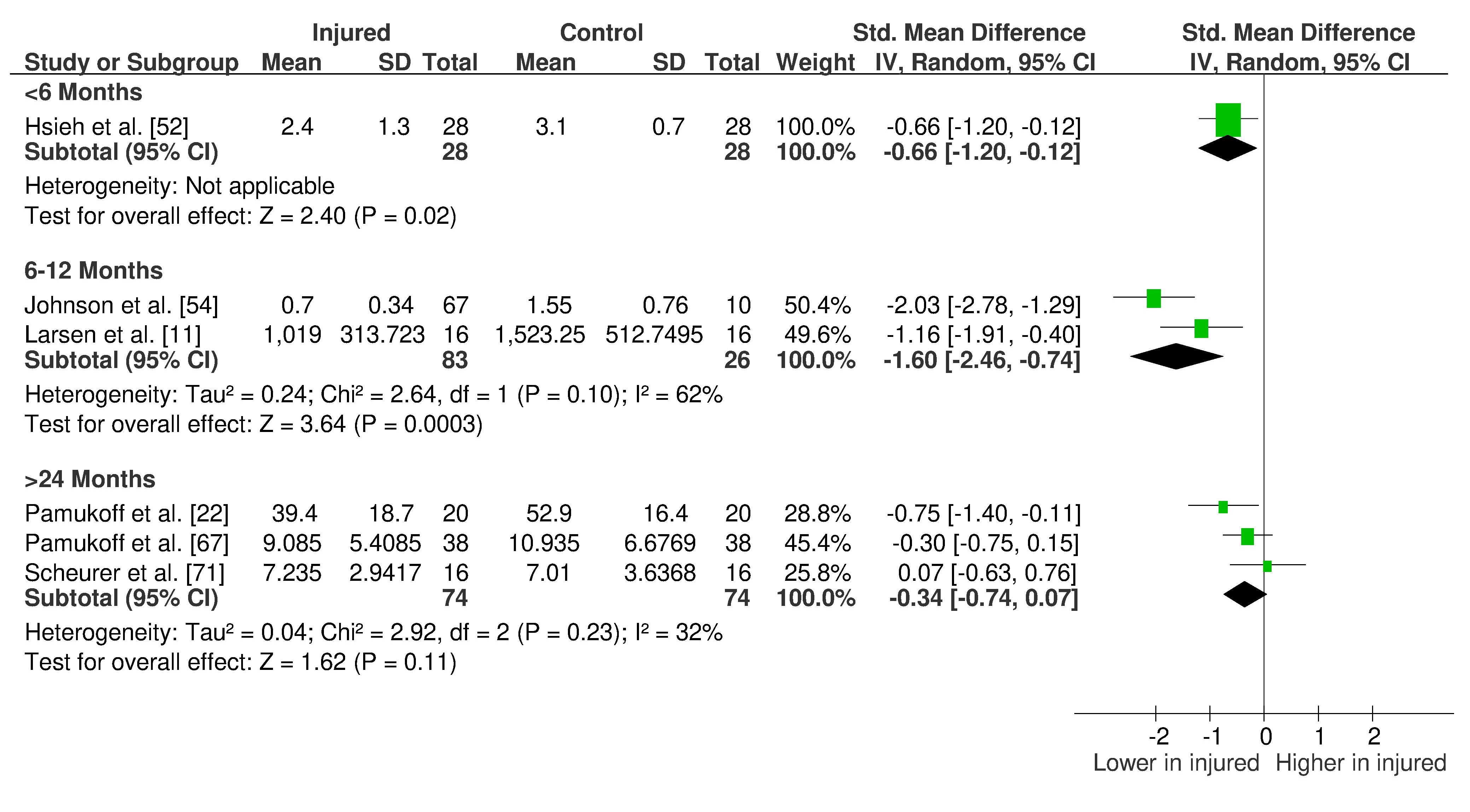


- 1. Quadriceps time to peak torque


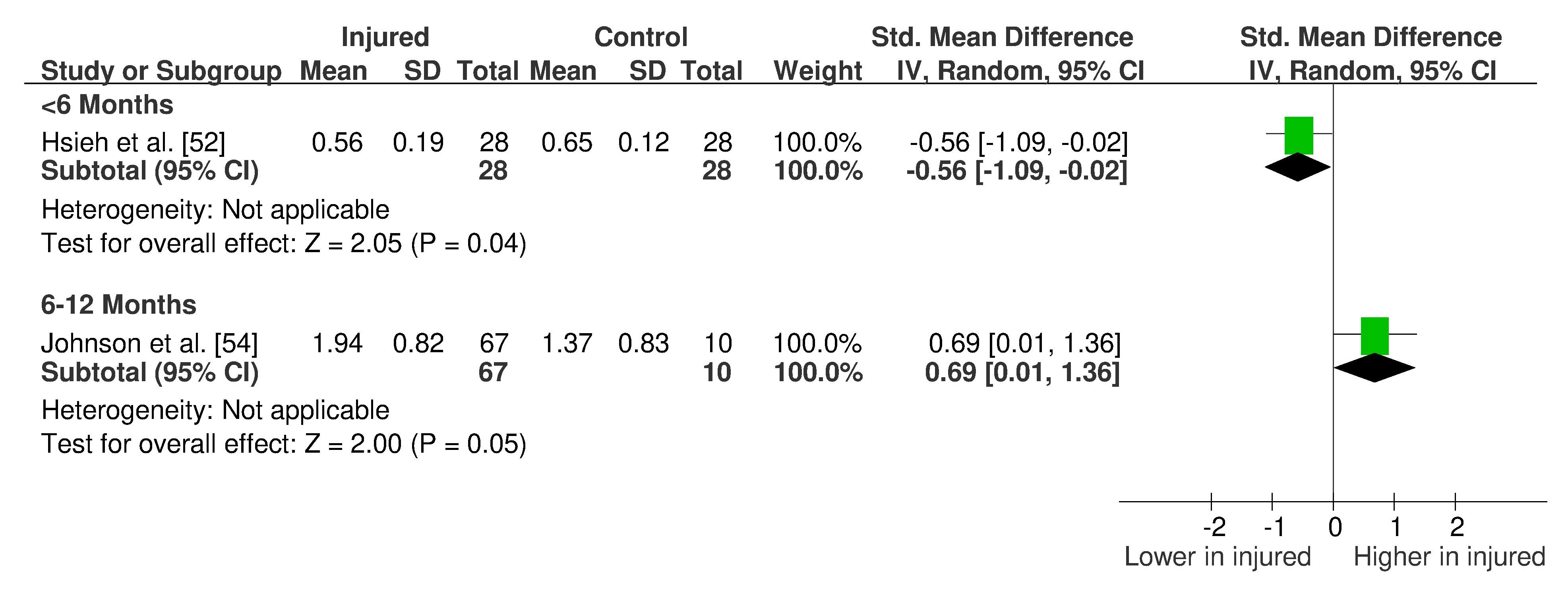


- 1. Quadriceps torque variability


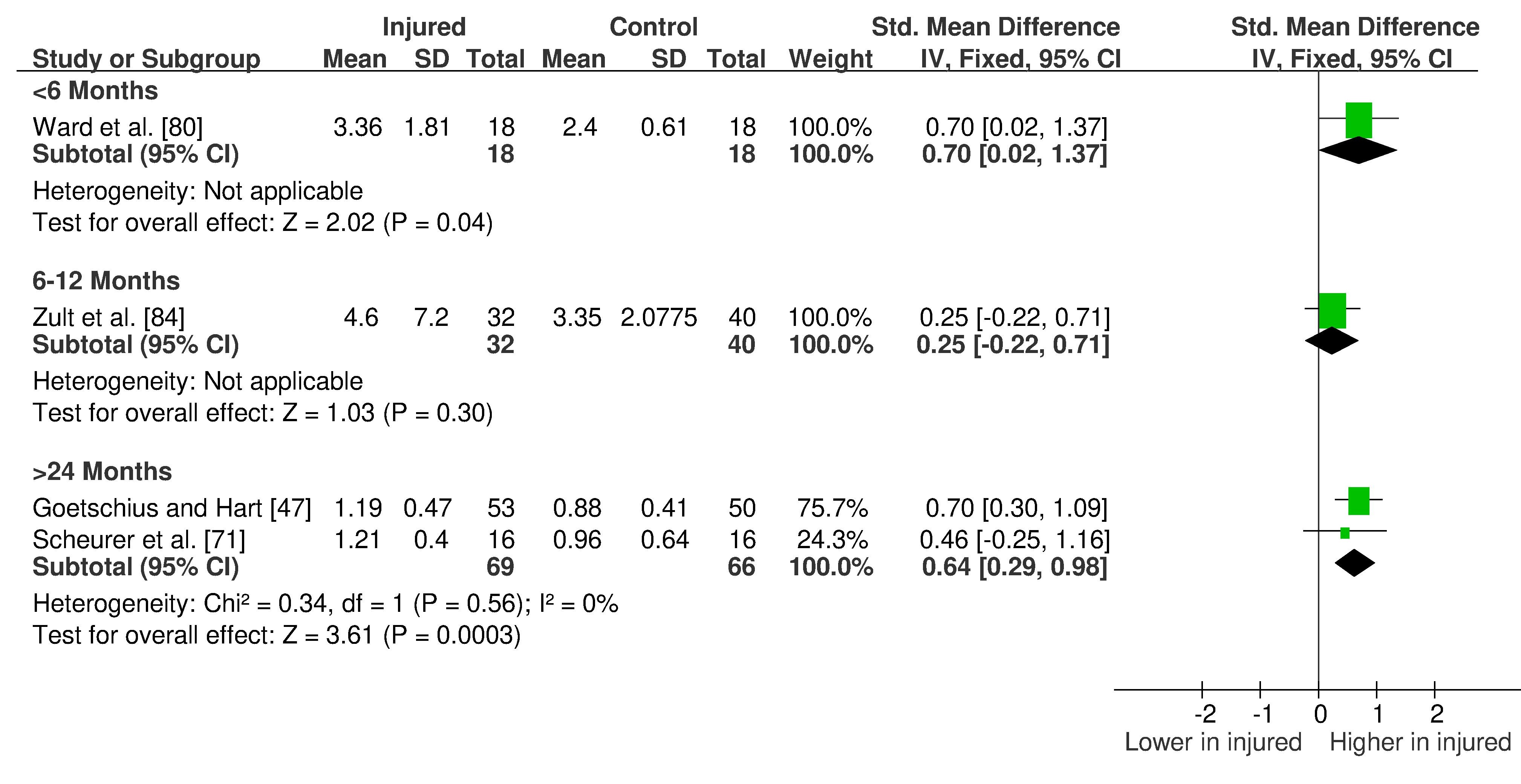


- 1. Quadriceps and hamstring electromechanical delay


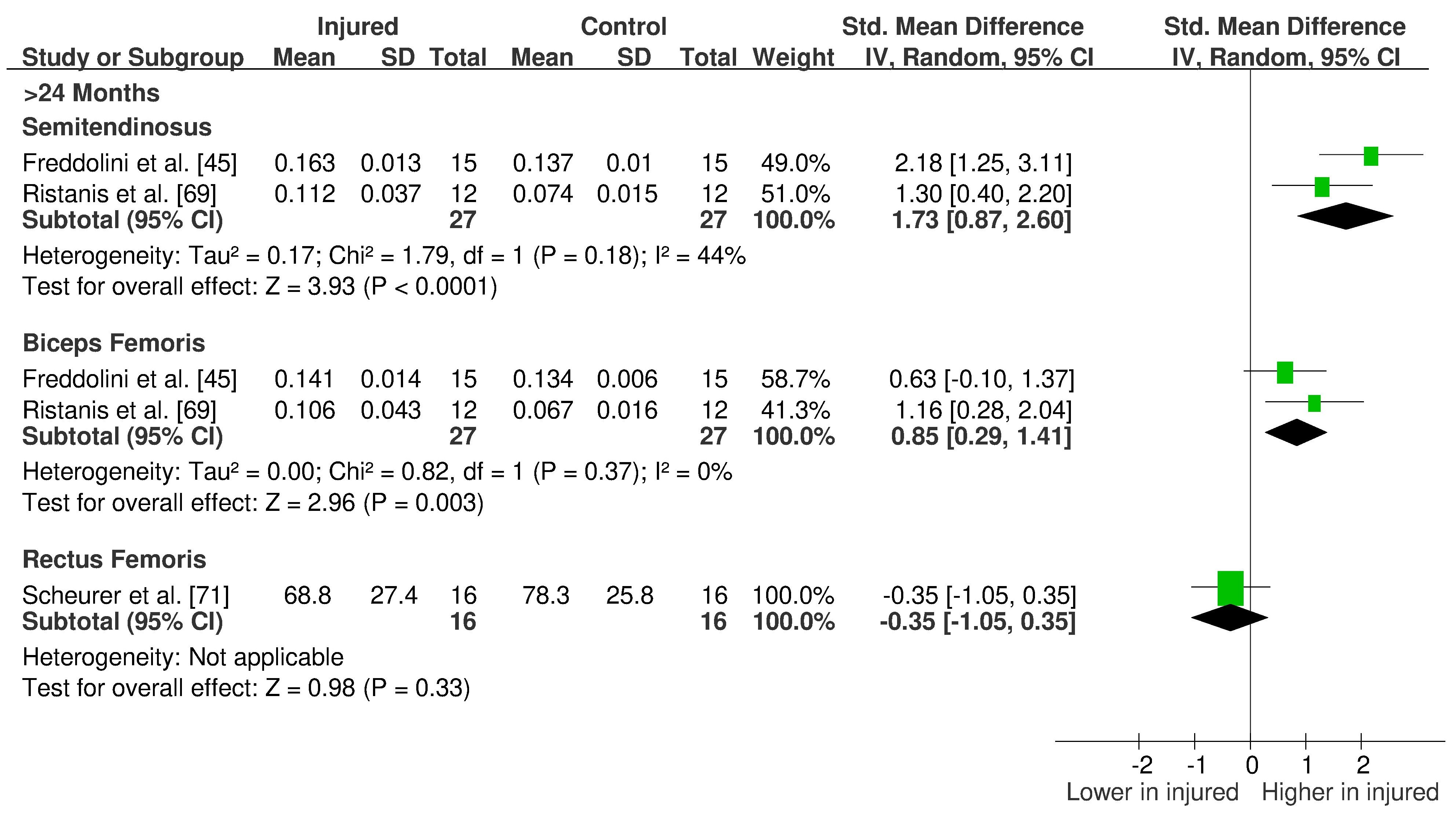


- 1. Hamstring isometric strength


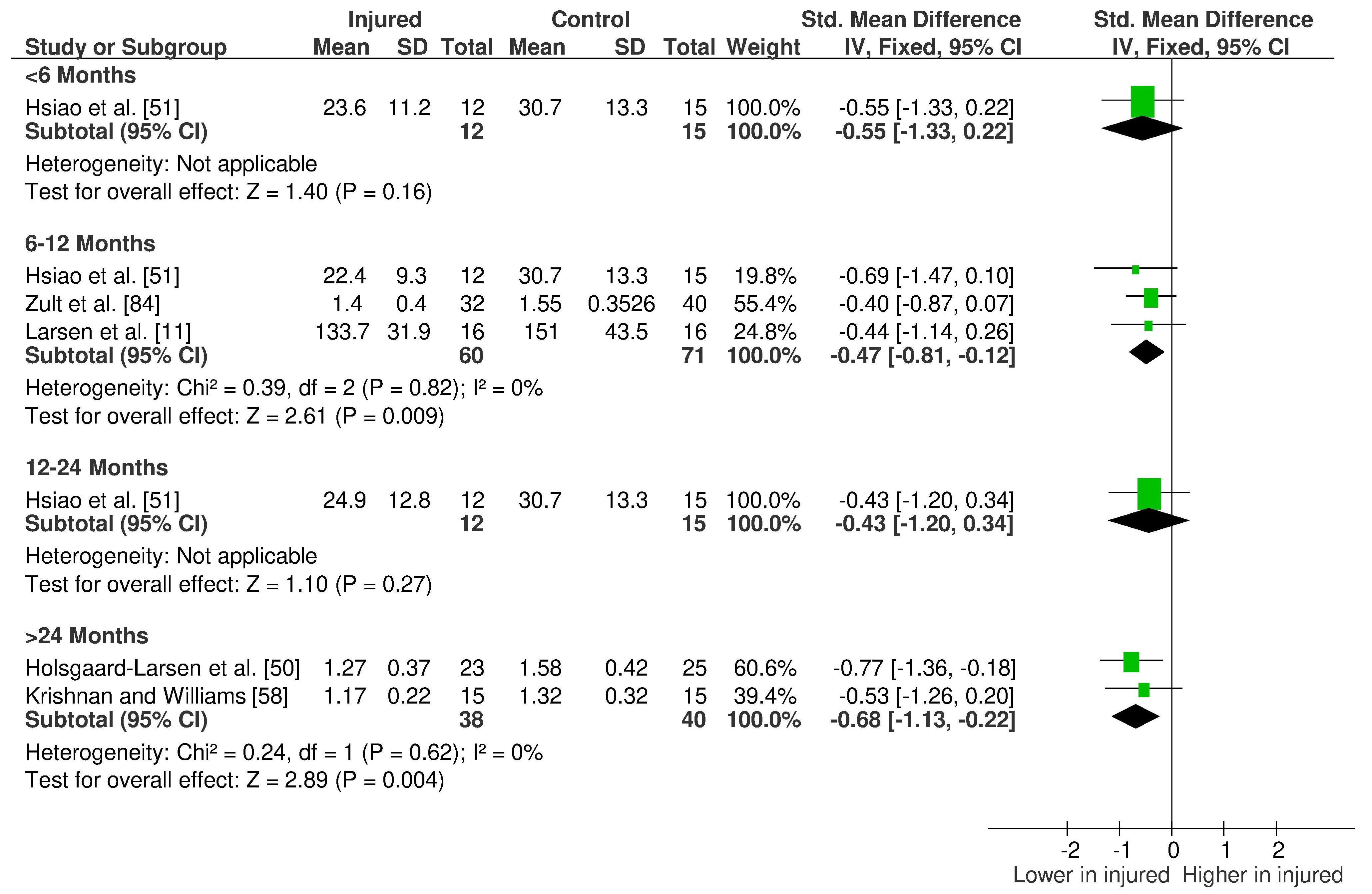


- 1. Hamstring fast concentric strength


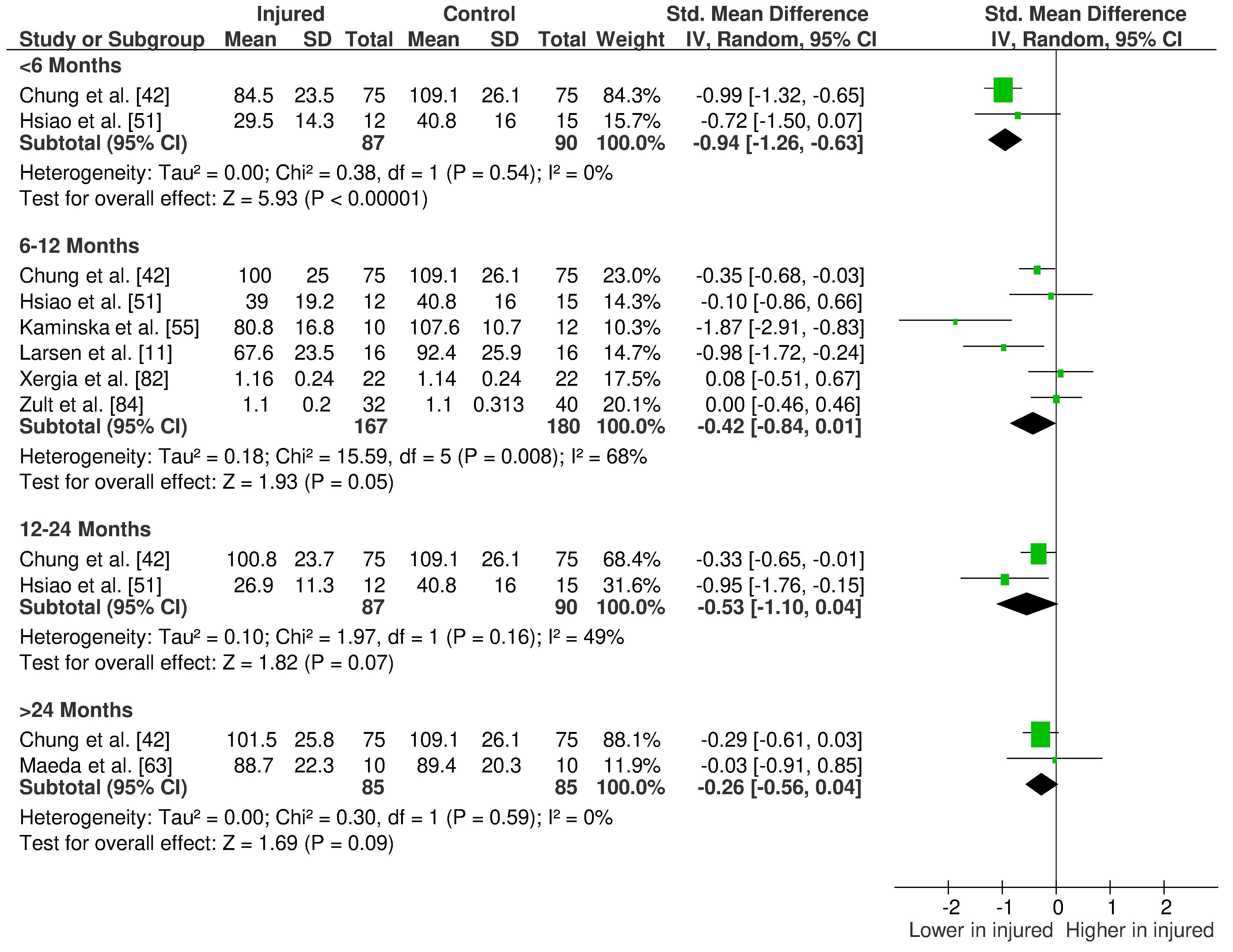


- 1. Hamstring eccentric strength


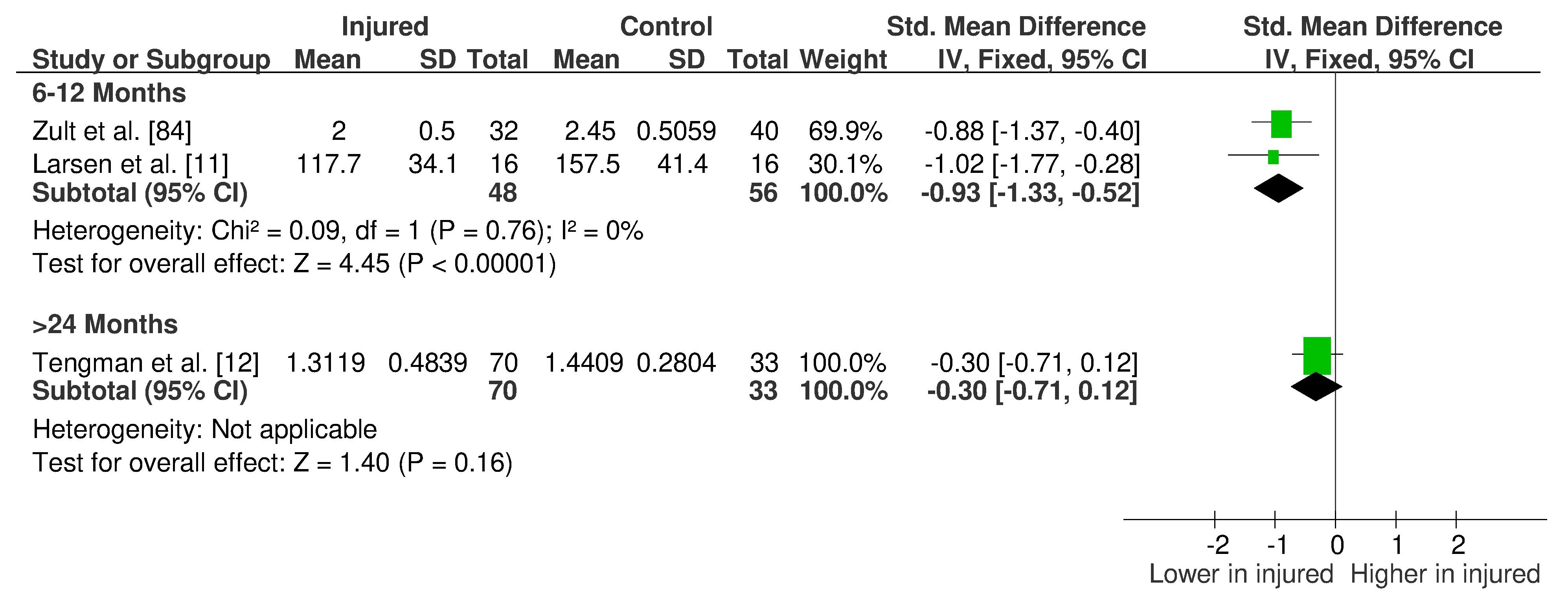


- 1. Hamstring rate of torque development


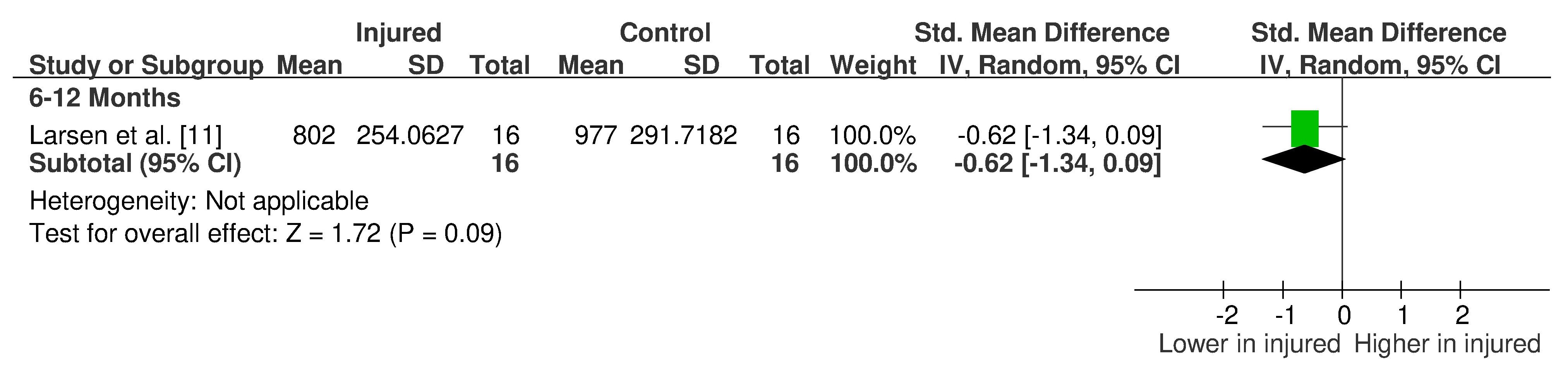


- 1. Hamstring: Quadriceps strength ratio


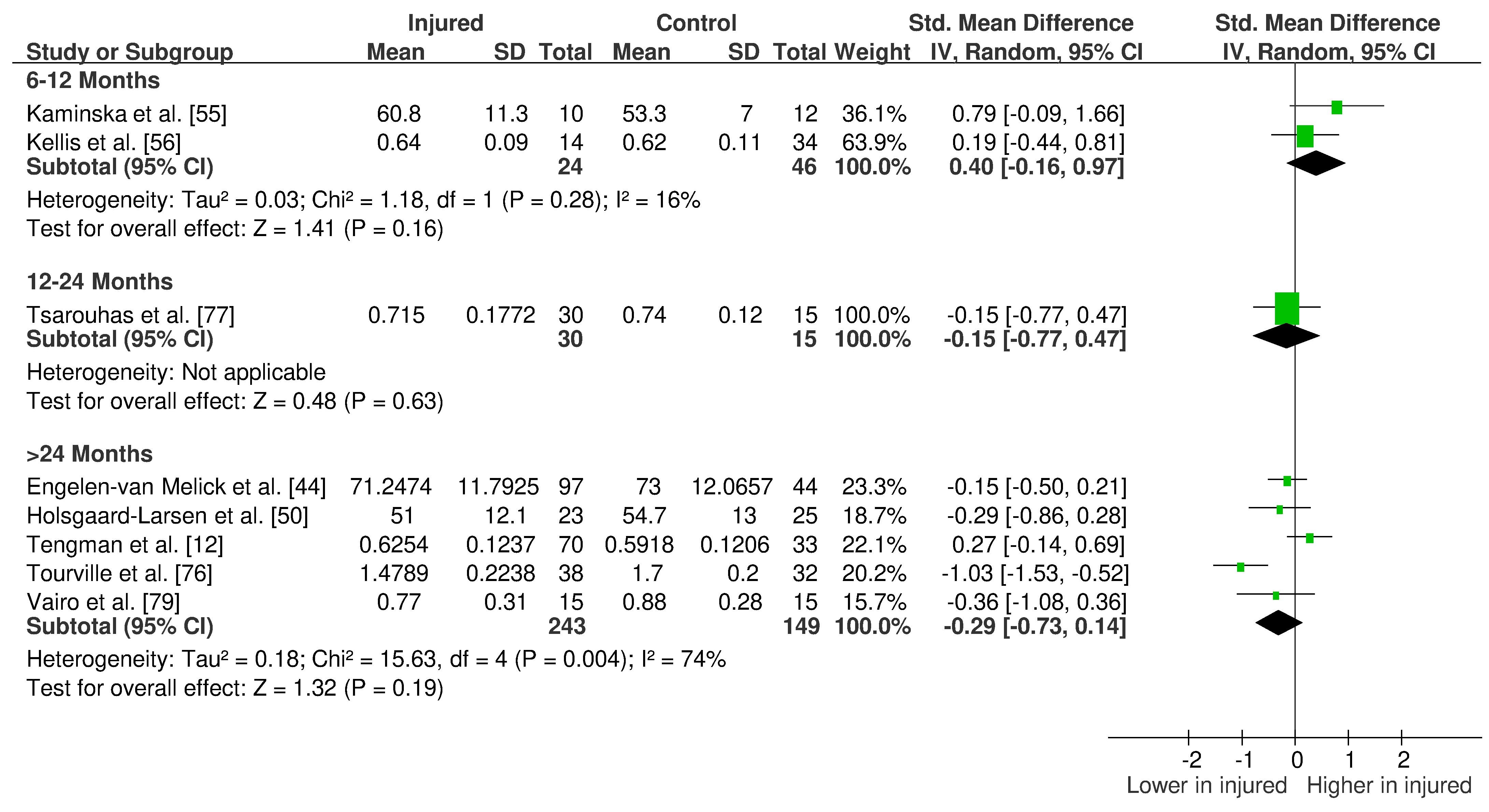


1. **Meniscus studies**
   1. Quadriceps isometric strength


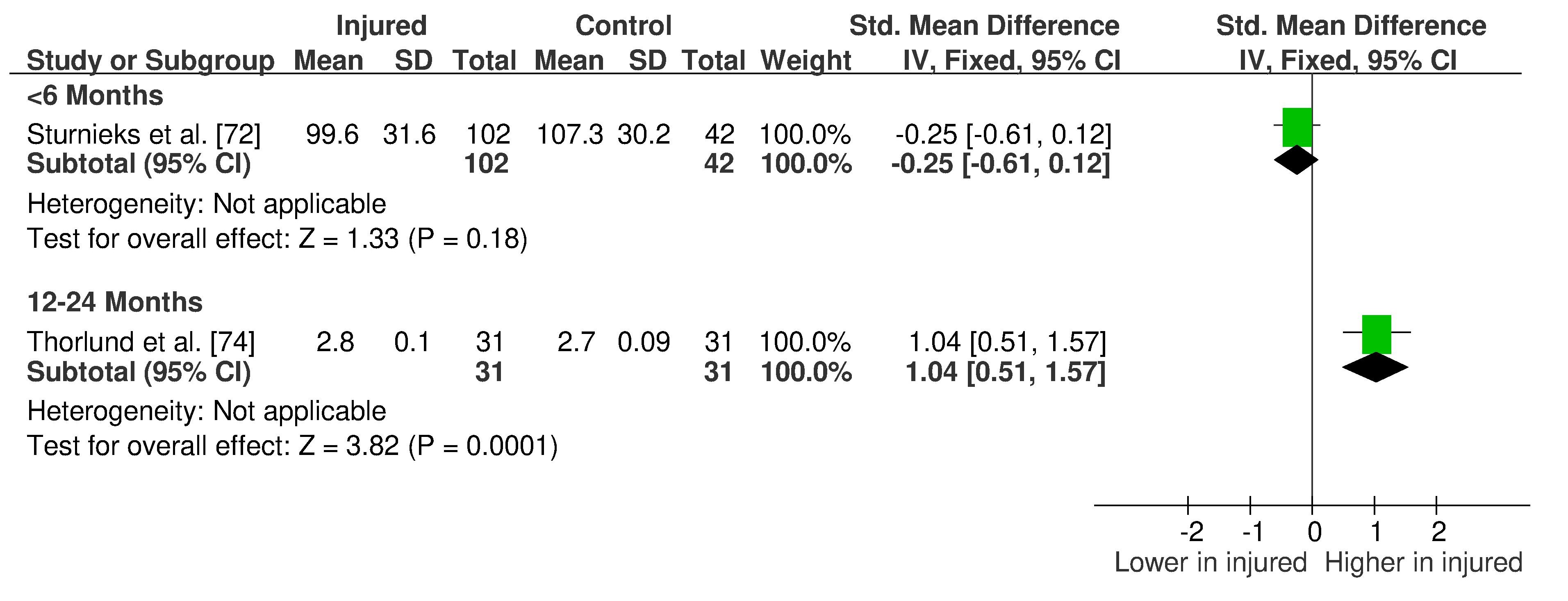


- 1. Quadriceps slow concentric strength


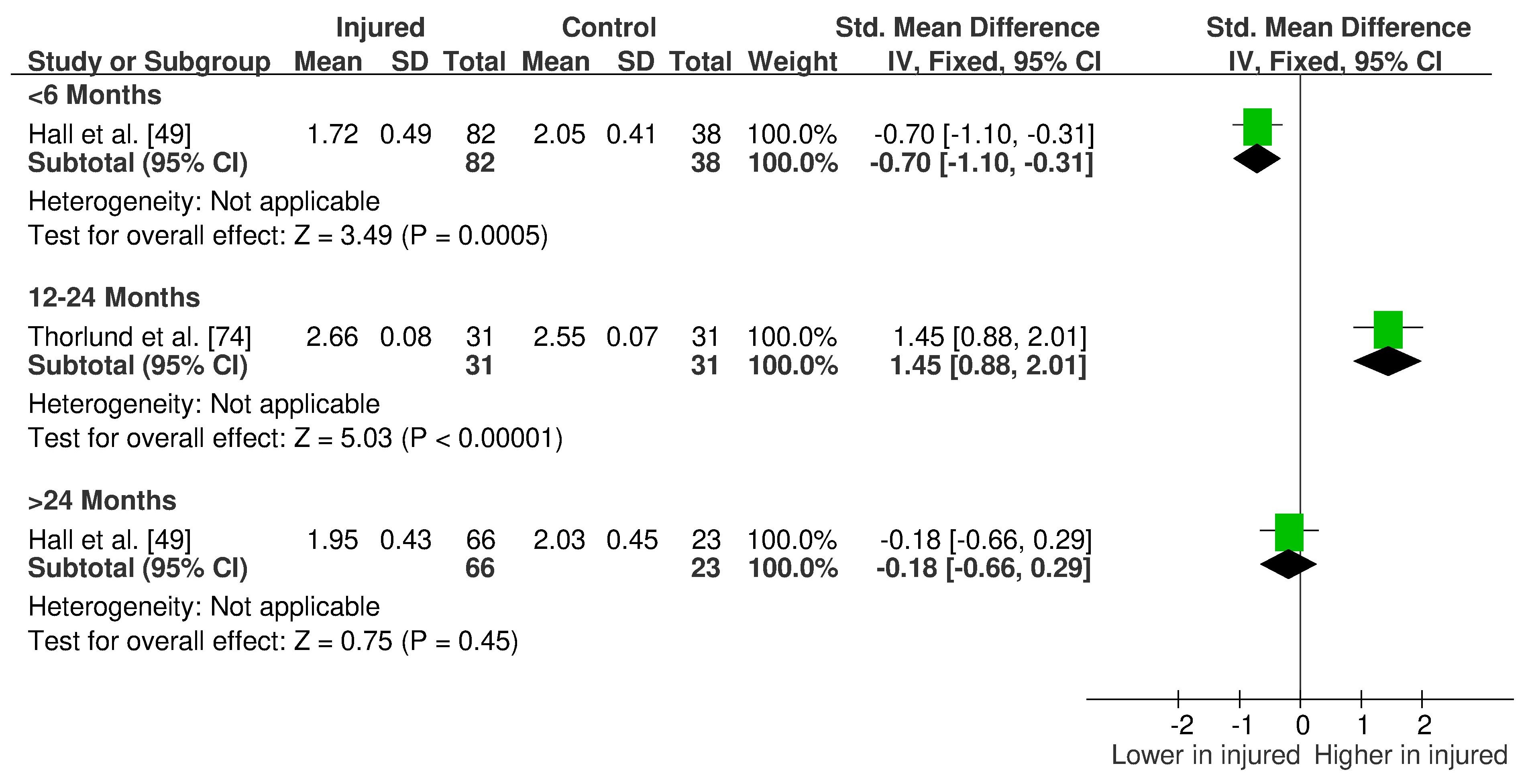


- 1. Quadriceps fast concentric strength


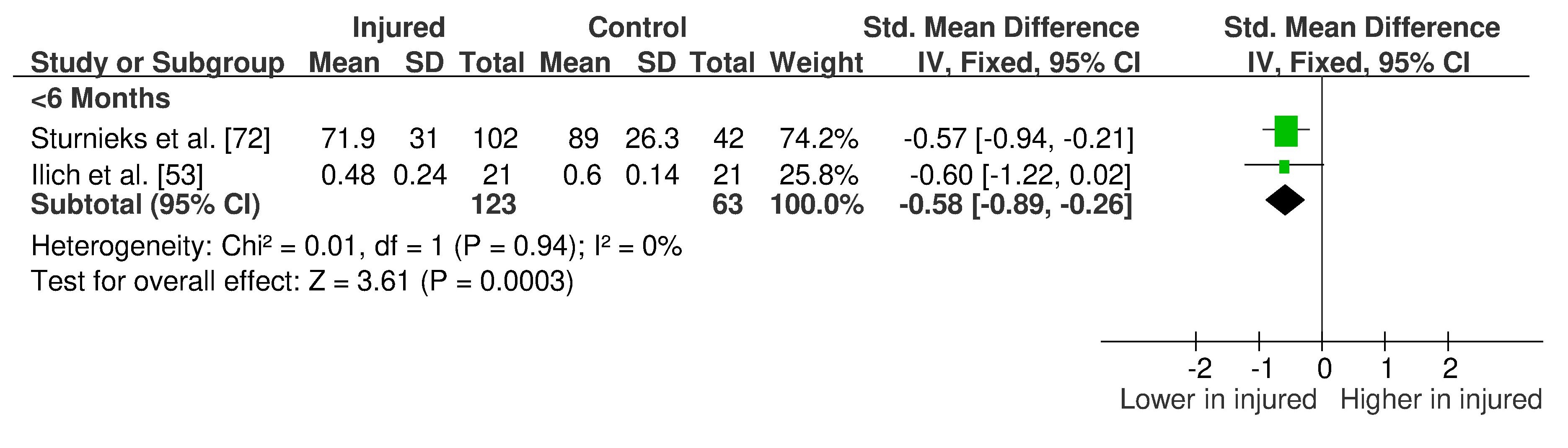


- 1. Quadriceps eccentric strength


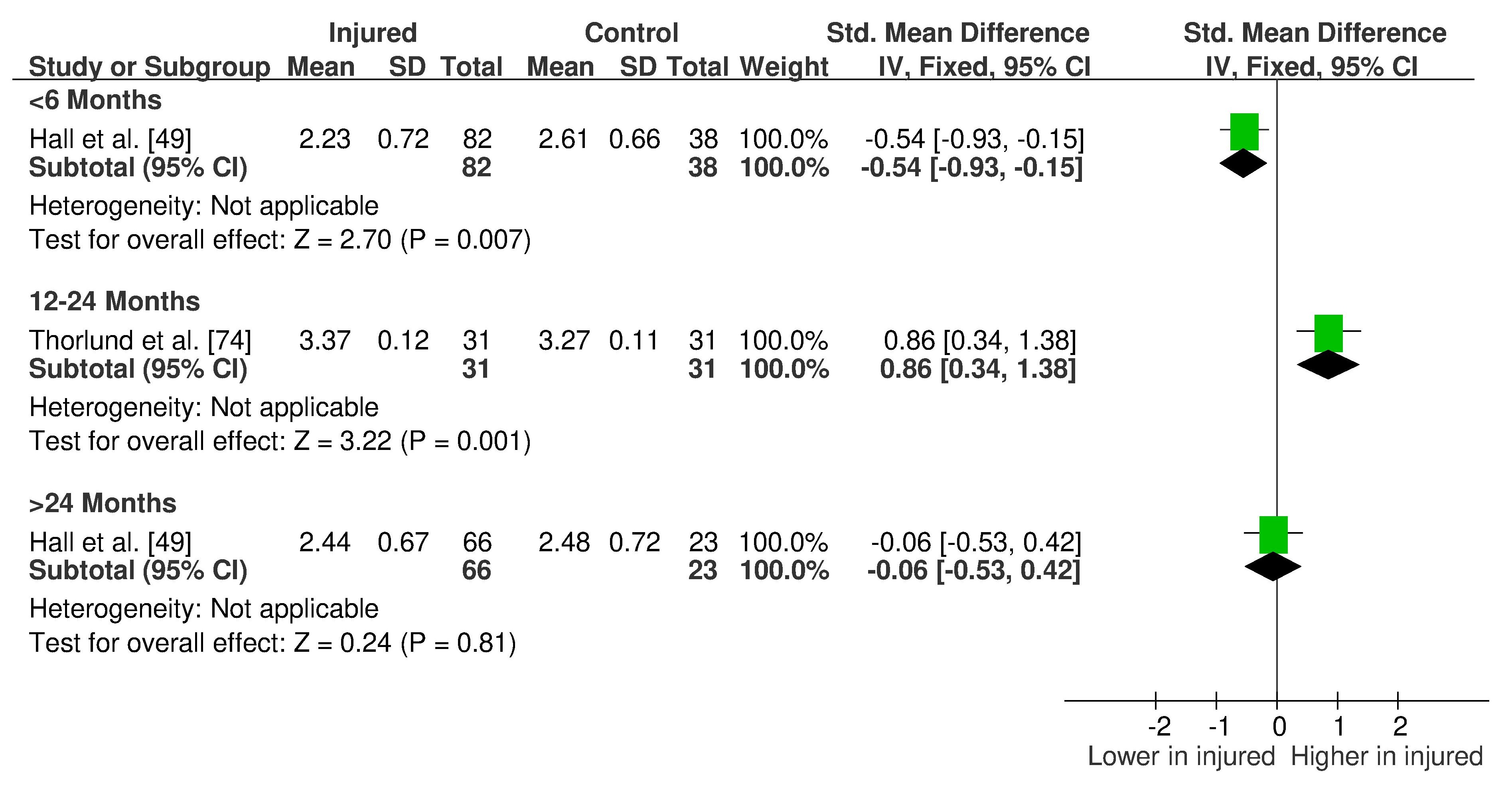


- 1. Quadriceps rate of torque development


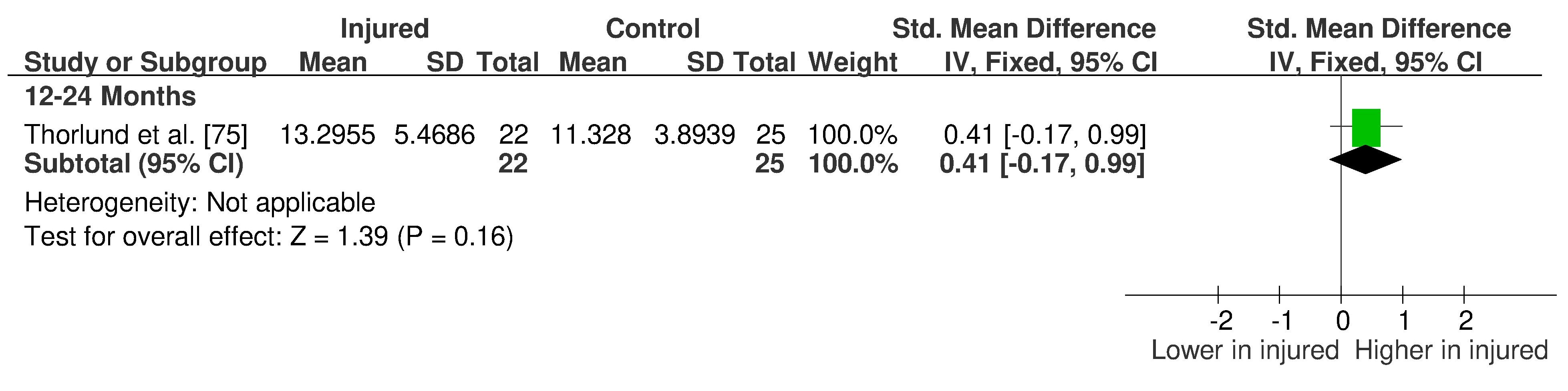


- 1. Hamstring isometric strength


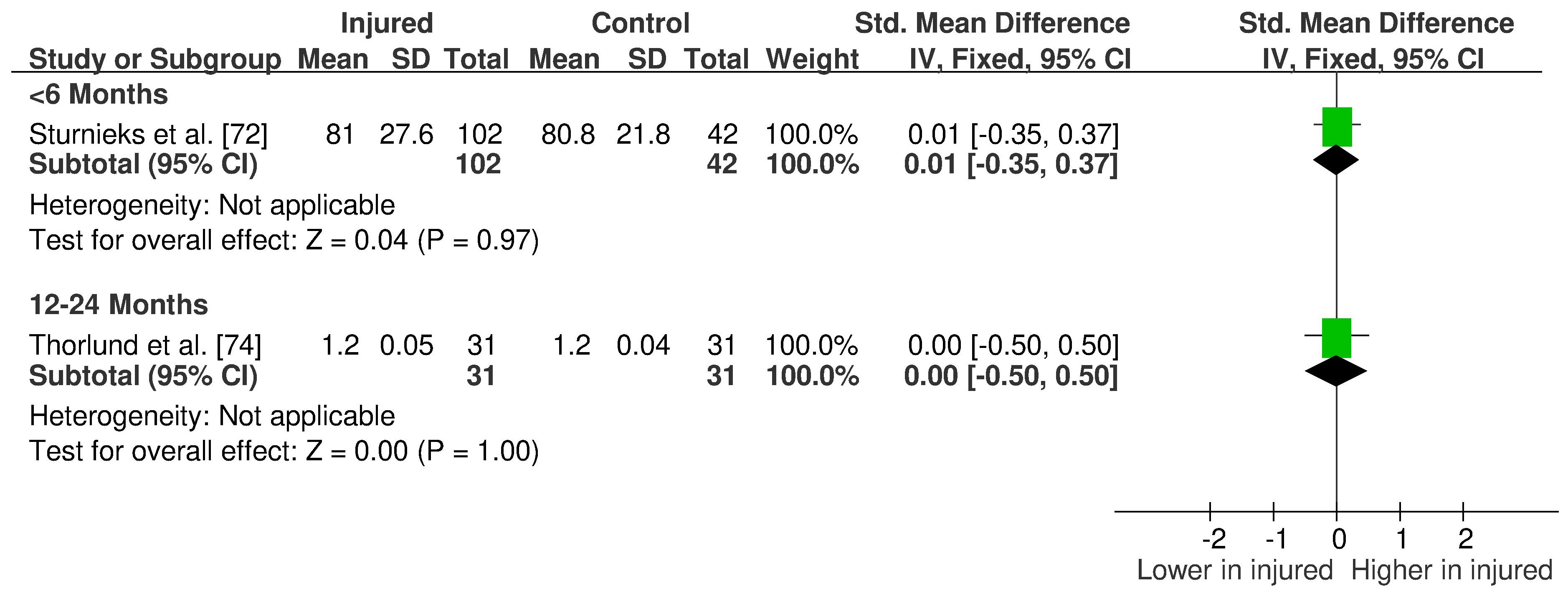


- 1. Hamstring slow concentric strength


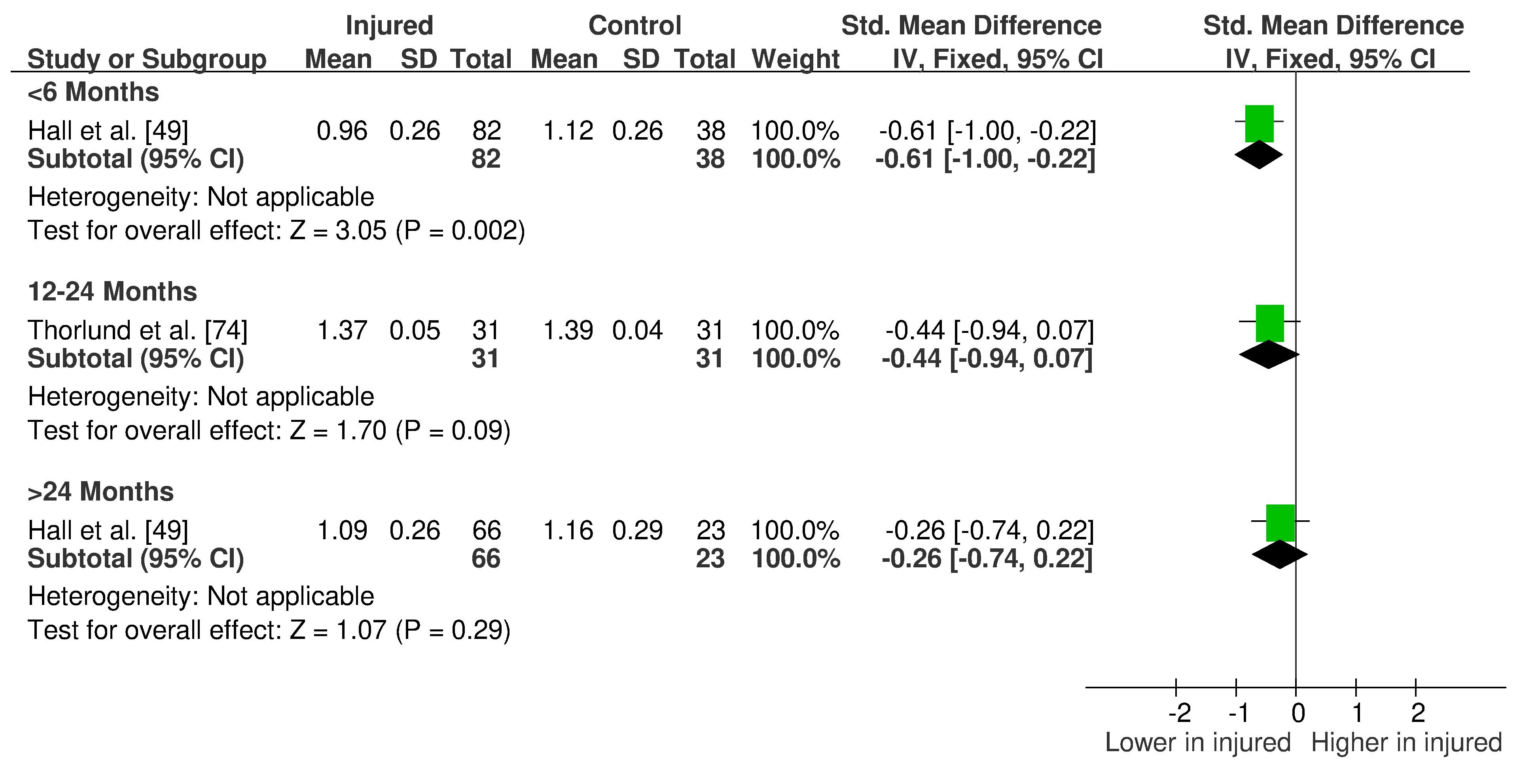


- 1. Hamstring fast concentric strength


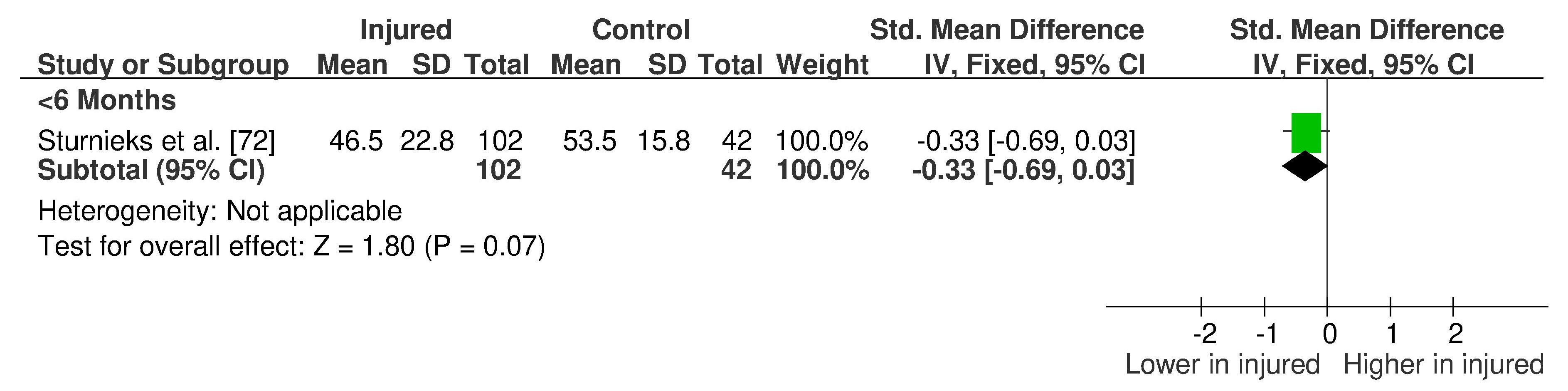


- 1. Hamstring eccentric strength


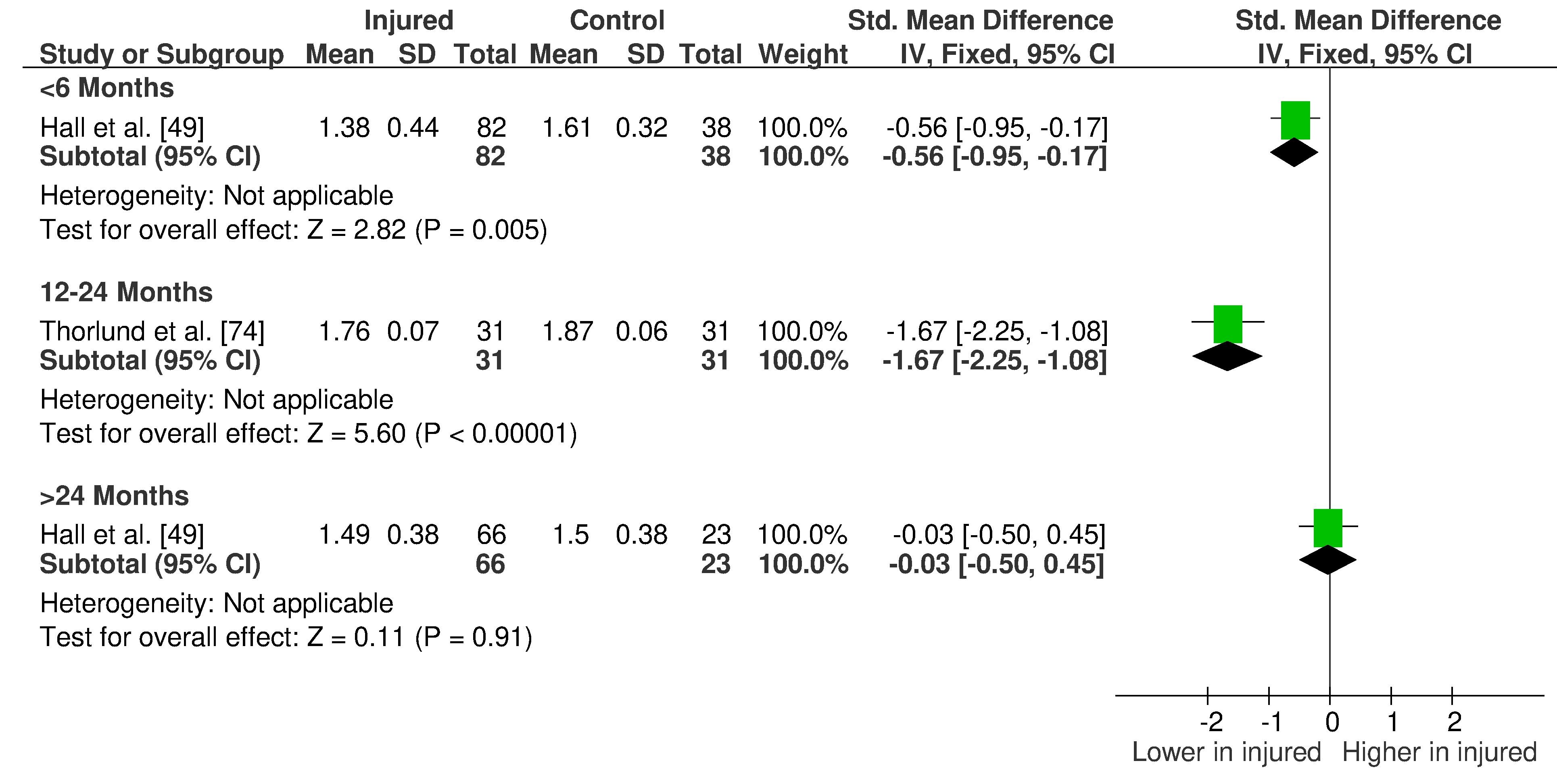


**DECLARATIONS**

**Funding**

Postgraduate studies of Ms Beyza Tayfur were sponsored by Turkish Ministry of National Education. The sponsors had no role in study design, data collection and analysis, decision to publish, or preparation of the manuscript.

**Conflicts of interest/Competing interests**

Beyza Tayfur, Chedsada Charuphongsa, Dylan Morrissey and Stuart Miller declare that they have no conflicts of interest relevant to the content of this review

**Ethics approval**

Not applicable

**Consent to participate**

Not applicable

**Consent for publication**

Not applicable

**Availability of data and material**

The data that support the findings of this study are available on request from the corresponding author [BT].

**Code availability**

Not applicable

**Authors' contributions**

Conception and design of the study: Beyza Tayfur, Dylan Morrissey and Stuart Charles Miller

Screening of the articles, data extraction, methodological quality ratings and data analysis: Beyza Tayfur and Chedsada Charuphongsa

First drafting of the manuscript: Beyza Tayfur

Critical revision of the manuscript: Dylan Morrissey and Stuart Charles Miller

All authors approved the version to be published.
